# Optimizing HIV retesting during pregnancy and postpartum in four countries: a cost‐effectiveness analysis

**DOI:** 10.1002/jia2.25686

**Published:** 2021-03-31

**Authors:** Julianne Meisner, D Allen Roberts, Patricia Rodriguez, Monisha Sharma, Morkor Newman Owiredu, Bertha Gomez, Maeve B de Mello, Alexey Bobrik, Arkadii Vodianyk, Andrew Storey, George Githuka, Thato Chidarikire, Ruanne Barnabas, Shiza Farid, Shaffiq Essajee, Muhammad S Jamil, Rachel Baggaley, Cheryl Johnson, Alison L Drake

**Affiliations:** ^1^ Department of Epidemiology University of Washington Seattle WA USA; ^2^ The Comparative Health Outcomes Policy & Economics Institute University of Washington Seattle WA USA; ^3^ Department of Global Health University of Washington Seattle WA USA; ^4^ Global HIV, Hepatitis and STI programme World Health Organization Geneva Switzerland; ^5^ Pan American Health Organization/World Health Organization Colombia Office Bogotá Colombia; ^6^ Department of Communicable Diseases and Environmental Determinants of Health Pan American Health Organization/World Health Organization Washington DC USA; ^7^ Global Fund to Fight AIDS, Tuberculosis and Malaria Geneva Switzerland; ^8^ Ukraine Country Office World Health Organization Kyiv Ukraine; ^9^ Clinton Health Access Initiative Boston MA USA; ^10^ National AIDS & STI Control Programme Nairobi Kenya; ^11^ HIV Prevention Programmes National Department of Health Pretoria South Africa; ^12^ Department of Medicine University of Washington Seattle WA USA; ^13^ HIV Section UNICEF New York NY USA

**Keywords:** maternal HIV retesting, elimination of maternal‐to‐child HIV transmission, EMTCT, prevention of maternal‐to‐child HIV transmission, PMTCT, cost‐effectiveness analysis, maternal and child health

## Abstract

**Introduction:**

HIV retesting during late pregnancy and breastfeeding can help detect new maternal infections and prevent mother‐to‐child HIV transmission (MTCT), but the optimal timing and cost‐effectiveness of maternal retesting remain uncertain.

**Methods:**

We constructed deterministic models to assess the health and economic impact of maternal HIV retesting on a hypothetical population of pregnant women, following initial testing in pregnancy, on MTCT in four countries: South Africa and Kenya (high/intermediate HIV prevalence), and Colombia and Ukraine (low HIV prevalence). We evaluated six scenarios with varying retesting frequencies from late in antenatal care (ANC) through nine months postpartum. We compared strategies using incremental cost‐effectiveness ratios (ICERs) over a 20‐year time horizon using country‐specific thresholds.

**Results:**

We found maternal retesting once in late ANC with catch‐up testing through six weeks postpartum was cost‐effective in Kenya (ICER = $166 per DALY averted) and South Africa (ICER=$289 per DALY averted). This strategy prevented 19% (Kenya) and 12% (South Africa) of infant HIV infections. Adding one or two additional retests postpartum provided smaller benefits (1 to 2 percentage point increase in infections averted versus one retest). Adding three retests during the postpartum period averted additional infections (1 to 3 percentage point increase in infections averted versus one retest) but ICERs ($7639 and in Kenya and $11 985 in South Africa) greatly exceeded the cost‐effectiveness thresholds. In Colombia and Ukraine, all retesting strategies exceeded the cost‐effectiveness threshold and prevented few infant infections (up to 31 and 5 infections, respectively).

**Conclusions:**

In high HIV burden settings with MTCT rates similar to those seen in Kenya and South Africa, HIV retesting once in late ANC, with subsequent intervention, is the most cost‐effective strategy for preventing infant HIV infections. In these settings, two HIV retests postpartum marginally reduced MTCT and were less costly than adding three retests. Retesting in low‐burden settings with MTCT rates similar to Colombia and Ukraine was not cost‐effective at any time point due to very low HIV prevalence and limited breastfeeding.

## 1 INTRODUCTION

Despite successful scale‐up of prevention of mother‐to‐child HIV transmission (PMTCT) programmes, an estimated 160 000 children worldwide became infected with HIV in 2018, with approximately 30% of these infections attributable to incident maternal infections occurring after the first antenatal care (ANC) visit [1, 2, 3, 4]. Compared to established infection, incident HIV infections acquired during pregnancy or postpartum increase the risk of mother‐to‐child HIV transmission (MTCT) nearly 10‐fold, due to both high viral load and the missed opportunity to detect these infections (and implement interventions to prevent MTCT) if initial testing occurs prior to transmission or seroconversion [5]. Identifying and treating maternal HIV infections is vital to achieving the UNAIDS 95‐95‐95 fast‐track targets for achieving low HIV incidence by 2030 [6].

Universal HIV testing for pregnant women has been found to be highly cost‐effective, even in very low HIV prevalence settings [7], and retesting during pregnancy and postpartum is a promising approach to reduce MTCT risk. The World Health Organization (WHO) has recommended retesting in the third trimester or during labour/delivery in high HIV prevalence settings since 2006, and retesting during breastfeeding since 2015 [8, 9]. Despite the promise of maternal HIV retesting, health and economic impacts and optimal timing and frequency are not well‐characterized [7, 10]. Modelling analyses in Uganda, South Africa and India have found retesting in late pregnancy, at delivery, or very early in the postpartum period to be cost‐effective [11, 12, 13]. However, recent scale‐up of ART coverage and HIV prevention interventions limit the relevance of these analyses, and in light of evidence that women in high burden settings have high HIV risk throughout the postpartum period [14, 15], postpartum retesting warrants investigation.

The HIV funding gap is projected to increase to US$7.2 billion below that needed to achieve 2030 fast‐track targets [16]. As countries strive to achieve the elimination of MTCT (EMTCT), policymakers must allocate limited resources efficiently. To provide guidance to countries deciding whether to implement maternal retesting, and if so the optimal frequency and time(s) to retest, we assessed the cost‐effectiveness of various strategies of HIV retesting during pregnancy, labour/delivery and postpartum in four countries representing a range of national HIV prevalence.

## 2 METHODS

### 2.1 Model structure and retesting strategies

We constructed a deterministic state‐transition model using Excel (Microsoft Corporation, Redmond, WA, USA) to reflect HIV disease progression, HIV testing and treatment during pregnancy and postpartum (54 total states: 6 maternal HIV stages stratified on 9 antenatal and postpartum periods) (Appendices S1 and S2). We chose to model four countries (South Africa, Kenya, Colombia and Ukraine) with varying geography and HIV prevalence and adequate data availability. Countries were classified based on WHO criteria as having high (>15%, South Africa), intermediate (5% to 15%, Kenya) and low national HIV prevalence (<5%, Colombia and Ukraine) [7]. We modelled the annual number of pregnant women in each country (or number of births as a proxy) and used weekly time steps from the start of pregnancy to 12 months postpartum. Infant disability‐adjusted life years (DALYs) and treatment costs were estimated over a 20‐year time horizon. We did not model infants explicitly; all cost and health events occurring after infant infection were estimated from model outputs. We evaluated six retesting scenarios varying the number and timing of retests (Table 1). We selected retest timing based on routine maternal and child health (MCH) visits/immunization schedules and country policies [10]. Current guidelines for retesting are as follows: South Africa – each ANC visit, labour and delivery and every three months while breastfeeding [17]; Kenya – third trimester, labour and delivery, six weeks and six months postpartum [18]; Ukraine – during pregnancy and labour and delivery only if high risk or unknown status [19]; Colombia – second and third trimester and labour and delivery [20]. We defined “late ANC” based on each country’s ANC visit schedule, median gestational age at ANC initiation and recommended HIV retesting interval. Late ANC corresponds to a gestational age in the third trimester in Kenya and South Africa and late second trimester in Colombia and Ukraine (Table 2a).

**Table 1:** Maternal HIV retesting scenarios

Scenario	Late ANC with catch‐up testing at delivery and 6 weeks postpartum	14 weeks	6 months (mid)	9 months (late)
1				
2	✓			
3	✓	✓		
4	✓		✓	
5	✓			✓
6	✓		✓	✓
7	✓	✓	✓	✓

Cells containing a ✓ denote a visit where retesting is offered. ANC, antenatal care visit. Catch‐up testing refers to testing at delivery for those who did not test in late ANC, and testing at six weeks postpartum for those who did not test at late ANC or delivery.

**Table 2a:** Model parameters

Parameter	Kenya	South Africa	Colombia	Ukraine
Population of pregnant women	1 631 479 [41]	1 100 699 [42]	346 409^b^	363 946 [19]
HIV risk				
HIV prevalence among pregnant women	6.1% [43]	31% [44]	0.4% [45]	0.7% [46]
Maternal HIV incidence rate (per person‐week)				
Prior to first ANC during pregnancy	0.000331 [47]	0.000227 [15]	0.00001	0.000002 [48]
Between first ANC and delivery	0.000331 [47]	0.000739 [15]	0.00002^a^	0.000004 [48]
Delivery to 6 weeks postpartum	0^a^	0^a^	0^a^	0^a^
6 weeks to 12 months postpartum	0.000269 [47]	0.0009 [15]	0.000023^a^	0.000003 [48]
Duration of acute maternal HIV infection (weeks)	9^a^	9^a^	9^a^	9^a^
HIV testing and prevention				
Test kit stock out	5%^a^	5%^a^	0%^a^	0%^a^
Test acceptance	84% [49]	98% [50]	89%^b^	97% [19]
Receive test results	98% [51]	98% [52]	100%^a^	100%^a^
HIV test sensitivity in early infection	67%^a^	67%^a^	67%^a^	67%^a^
HIV test sensitivity in chronic infection	100% [53]	100% [53]	100% [53]	100% [53]
HIV test specificity	98.9% [53]	98.9% [53]	98.9% [53]	98.9%[53]
MTCT rate per week, acute maternal infection	0.005 to 0.029 [54, 55, 56, 57, 58]	0.005 to 0.029 [54, 55, 56, 57, 58]	0.005 to 0.029 [54, 55, 56, 57, 58]	0.005 to 0.029 [54, 55, 56, 57, 58]
MTCT rate per week, chronic maternal infection	0.0005 to 0.023 [54, 55, 56, 57, 58]	0.0005 to 0.023 [54, 55, 56, 57, 58]	0.0005 to 0.023 [54, 55, 56, 57, 58]	0.0005 to 0.023 [54, 55, 56, 57, 58]
Maternal PrEP use	0%^a^	0%^a^	0%^a^	0%^a^
Health care visits				
Attend first ANC	96% [59]	94% [52]	97%^b^	99% [18]
Attend late ANC	93% [59]	78% [52]	88%^b^	90%^a^
Facility delivery	62% [59]	96% [52]	99%^b^	99% [60]
Attend postnatal MCH visits				
6 weeks	96% [59]	90%^b^	92% [61]	99%^a^
14 weeks	88% [59]	73%^b^	87% [61]	93%^a^
6 months	87% [59]	86%^b^	90% [61]	96%^a^
9 months	85% [59]	62%^b^	89% [61]	95%^a^
First ANC (gestational age in weeks)	22 [23]	18^b^	15^b^	10^a^
Late ANC (gestational age in weeks)	33^a^	36^a^	24^a^	28^a^
Delivery (gestational age in weeks)	39^a^	39^a^	39^a^	39^a^
Early postpartum (weeks)	6^a^	6^a^	6^a^	6^a^
Mid postpartum (weeks)	26^a^	26	26^a^	26^a^
Antiretroviral coverage				
Maternal ART use	91% [4]	87% [4]	88% [62]	95% [46]
Virally suppressed^c^	88% [63]	72% [64]	88% [63]	88% [63]
Weekly risk of ART dropout	0.33% [22]	0.33% [22]	0.33% [22]	0.33% [22]
HIV‐exposed infants receiving ARVs	94% [23]	99%^b^	96%^b^	98%^a^
HIV‐infected infants receiving ART^d^	61% [43]	63% [78]	57.9% [62]	95% [46]
Breastfeeding practices among HIV infected women				
Not breastfeeding in early postpartum (0 to 6 weeks)	2.5%^b^	34% [66]	95% [62]	95%^a^
Not breastfeeding in mid postpartum (6 weeks to 6 months)	21%^a^	45% [66]	98% [62]	99%^a^
Not breastfeeding in late postpartum (6 to 12 months)	33%^b^	63% [66]	98% [62]	99%^a^
Maternal mortality rate (per person‐week)				
During pregnancy	0.0001 [29]	0.0001 [29]	0.00002 [29]	0.00002 [29]
Delivery through 6 weeks postpartum	0.0006 [68]	0.0002 [68]	0.0001 [68]	0.00003 [68]
6 weeks to 12 months postpartum	0.0001 [29]	0.0001 [29]	0.00002 [29]	0.00002 [29]
Neonatal/infant mortality (per person‐week) and survival probabilities				
Neonatal mortality, birth‐6 weeks	0.0049 [69]	0.0029 [69]	0.0018 [69]	0.0011 [69]
Infant mortality, >6 weeks to 12 months	0.00031 [69]	0.00025 [69]	0.00009 [69]	0.00006 [69]
Survival to 1 year, HIV−	96% [29]	97% [29]	99% [29]	99% [29]
Survival to 1 year, HIV + on ART	96% [29]	97% [29]	99% [29]	99% [29]
Survival to 1 year, HIV + not on ART	65% [30]	65% [30]	65% [30]	65% [30]

ANC, antenatal care; ARV, antiretroviral prophylaxis; ART, antiretroviral therapy; MCH, maternal and child health; MTCT, mother‐to‐child transmission; PrEP, pre‐exposure prophylaxis.

^a^ Indicates assumption

^b^ indicates in‐country source

^c^ among women on ART

^d^ based on the percent of infants with early infant diagnosis.

### 2.2 Model assumptions and parameterization

Model parameters (Table 2a; Appendix S3) were derived from published literature (through April 2020), expert opinion, or assumptions if data were not available. As our model simulates a hypothetical population not corresponding to a specific year, for all parameters we used the most up‐to‐date value available. HIV testing in our model requires visit attendance, tests in stock, test acceptance and receipt of results. In the base case scenario, HIV testing is offered only once at the first ANC, which is assumed to occur at a country‐specific gestational age. To reflect a setting in which retesting at delivery or six weeks postpartum would only occur if women had not retested previously, we modelled catch‐up retesting at delivery to be conditional on the probability of not retesting at late pregnancy, and retesting at six weeks postpartum to be conditional on the probability of not retesting in late ANC or delivery.

We assumed no retesting between visits, no viral suppression during recent (acute) infection, no repeat or multiple pregnancies and no foetal losses. We assumed all women diagnosed HIV‐positive were ART eligible [21] and based ART initiation and viral suppression rates on country‐specific estimates. We assumed a constant weekly risk of ART discontinuation resulting in 74% retention by one year postpartum [22]. MTCT depended on pregnancy/postpartum period; maternal disease stage, use of ART and viral load suppression; infant use of antiretroviral prophylaxis (ARVs); and breastfeeding practices (Appendix S3). Infant HIV testing was only reflected in the probability an infected infant received ART, taken as the product of the probability of testing and treatment uptake. We assumed no pre‐exposure prophylaxis (PrEP) use in the main analyses. The model was validated against estimated MTCT rates from each country [18, 23, 24, 25] (Appendix S4).

### 2.3 Health outcomes

Modelled health outcomes included infant HIV infections, deaths and DALYs. In addition to percent of total infant infections averted by each scenario, we calculated the percent of maximum possible infant infections averted relative to no retesting by excluding infant infections from women diagnosed with HIV prior to pregnancy, as these women would neither be eligible for nor benefit from additional HIV testing.

### 2.4 Costs and cost‐effectiveness

We estimated costs from a healthcare system perspective, including costs for maternal retesting, maternal ART, infant ARV prophylaxis and infant ART (Table 2b, Appendix S2). Costs and resource utilization were obtained from the published literature, a time‐motion study in Kenya (Appendix S5), in‐country experts, or assumptions (Table 2b, Appendix S2). Unit costs were applied to testing and treatment/prophylaxis as women transitioned though model states. Costs were adjusted to 2017 USD [26].

**Table 2b:** Costs

Parameter	Kenya	South Africa	Colombia	Ukraine
Third generation rapid screening per woman	2.64^a^	7.72^a^, ^b^	6.68^a^, ^b^	3.99^a^, ^b^
True‐positive screening tests per woman†	3.68^a^	11.39^a^, ^b^	8.53^a^, ^b^	4.18^a^, ^b^
False‐positive screening tests per woman†	26.39^a^	34.17^a^, ^b^	74.83^a^, ^b^	19.80^a^, ^b^
Maternal ART, per week	4.86 [71]	4.79 [72]	18.89^b^	32.84 [73]
Infant ARV prophylaxis (total cost)	2.32 [74]	3.82 [74]	52.10^b^	4.00^b^
Maternal PrEP, per week	6.19 [75]	6.19 [75]	18.89^b^	19.38 [76]
Infant ART, per week (birth to 2 weeks)	6.73 [77]	5.46 [74]	18.89^b^, ^c^	32.84^c^
Infant ART per week (2 weeks to 1 year)	6.73 [77]	5.46 [74]	18.89^c^	32.84^c^

All costs provided in 2017 USD and include labour and supplies. ART, antiretroviral therapy; ARV, antiretrovirals; PrEP, pre‐exposure prophylaxis.

^a^ Micro‐costing estimate

^b^ in‐country source

^c^ assumption.

Weekly maternal ART costs were applied to women receiving ART and modelled through 12 months postpartum. We modelled PrEP in scenario implementation analyses, with women testing HIV‐negative being eligible for PrEP and incurring PrEP‐related costs. Costs of infant prophylaxis were applied as a one‐time cost to women diagnosed by delivery. Weekly ART costs were applied to HIV‐infected infants throughout the 20‐year time horizon. Both costs and DALYs were discounted at 3% annually [27].

We converted infant infections and deaths into DALYs [28]. We estimated DALYs through 20 years of life using country‐specific WHO life tables and age‐ and HIV status‐specific mortality estimates from published literature (Appendices S2 and S3) [29, 30]. We choose a time horizon of 20 years for comparability with other HIV cost‐effectiveness analyses. We calculated the incremental cost‐effectiveness ratio (ICER) as the change in costs divided by the change in health outcomes compared with the next best alternative. Strategies that were more costly and less effective (“strongly dominated”) or less costly and less cost‐effective (“weakly dominated”) than an alternative strategy were considered inefficient [31] and eliminated from the calculations. We used country‐specific cost‐effectiveness thresholds based on the estimated opportunity cost of health investment foregone. We adopted thresholds of $500 (Kenya) [32, 33]; $750 (South Africa), derived in [34] as in Phillips et al. [35]; $1000 (Ukraine) [36] and $3000 (Colombia) [37] per DALY averted. We followed the Consolidated Health Economic Evaluation Reporting Standards (CHEERS) in preparing this manuscript [38].

### 2.5 Scenario implementation and uncertainty analyses

We estimated the sensitivity of Scenario 2 results in Kenya and South Africa to uncertainty in key parameters by modelling 20% relative increases and decreases (bounded by 0 and 1 for probabilities). We also evaluated the sensitivity of model Scenarios 2 and 3 in each country to reductions in maternal HIV incidence and prevalence, and introduction of PrEP. We modelled PrEP use among 5%, 10% and 15% of all HIV‐negative women. We additionally modelled a combination scenario of 15% PrEP use and 20% reduction in both maternal HIV prevalence and incidence. Last, to assess whether the benefits of retesting would change with an optimized HIV care cascade, we estimated the percentage of infections averted with retesting assuming 100% MCH attendance, test coverage, treatment initiation, retention, viral suppression and infant ARV prophylaxis coverage (Appendix S6).

### 2.6 Ethical statement

This research involved no identifiable data, and as such does not constitute human subjects research.

## 3 RESULTS

**Figure 1:**
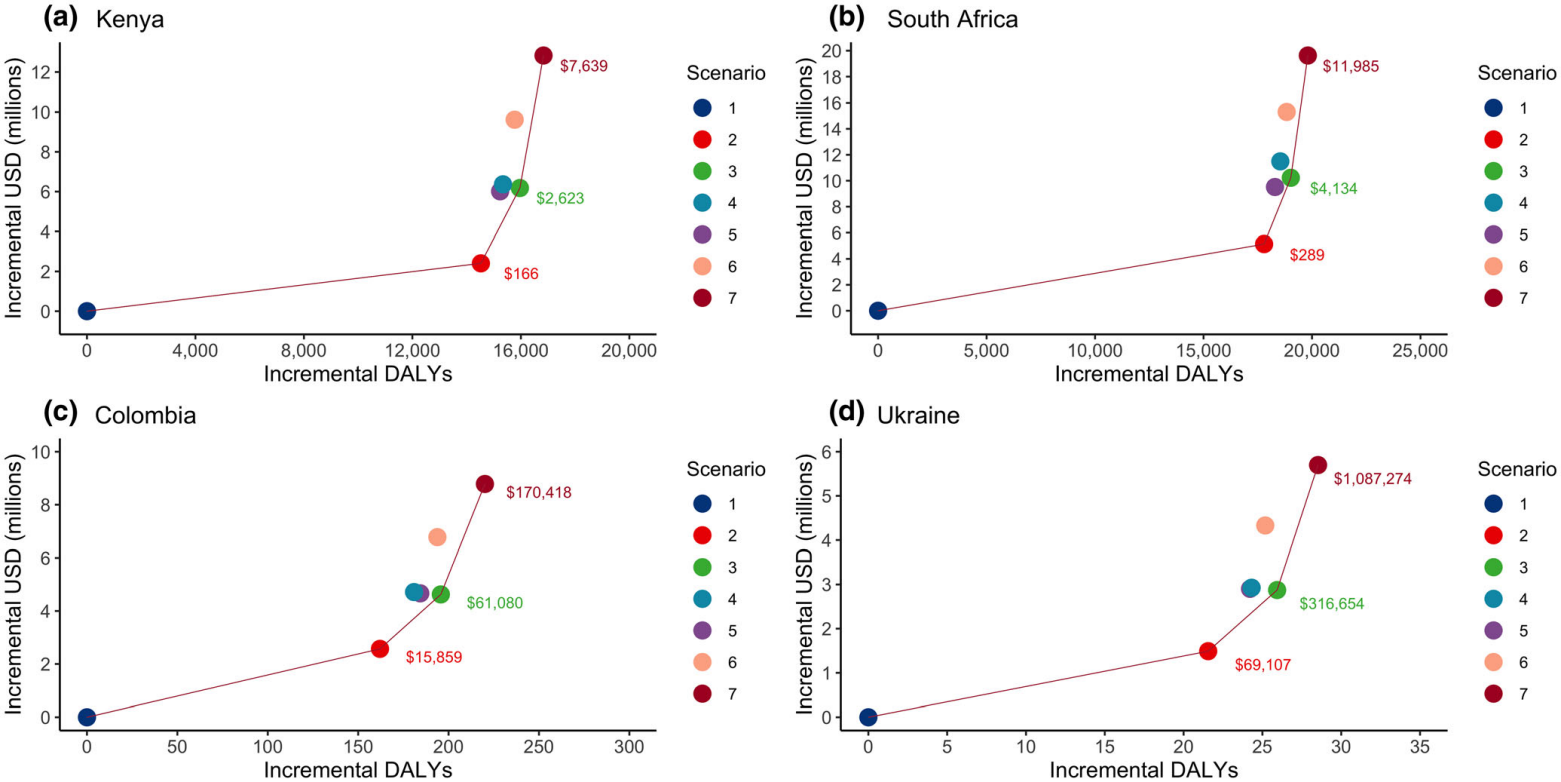
Efficiency frontier. presenting the incremental disability‐adjusted life years (DALYs) averted and costs (in 2017 USD) for six retesting scenarios relative to Scenario 1 (no retesting) in (a) Kenya, (b) South Africa, (c) Colombia, (d) Ukraine. The solid line indicates scenarios that are not dominated by other scenarios, where “dominated” indicates a scenario is more costly and less effective. The text indicates incremental cost‐effectiveness ratios for the non‐dominated scenarios compared to the next least‐costly scenario connected by a solid line. Scenario 1: no retesting; Scenario 2: retesting in late ANC/delivery/6 weeks postpartum; Scenario 3: scenario 2 plus retesting at 14 weeks postpartum; Scenario 4: scenario 2 plus retesting at six months postpartum; Scenario 5: scenario 2 plus retesting at nine months postpartum; Scenario 6: scenario 2 plus retesting at both six and nine months postpartum; Scenario 7: scenario 2 plus retesting every three months postpartum.

Model results are presented in Table 3 and Figure 1. In Kenya and South Africa, HIV retesting among women of HIV‐negative or unknown status in late ANC, with catch‐up testing through six weeks postpartum for women not tested in late ANC, is projected to prevent 19% and 12% of infant infections respectively. In both countries, ICERs associated with this retesting strategy ($166 and $289 per DALY averted for Kenya and South Africa respectively) fell below the cost‐effectiveness thresholds. In these countries, adding one or two additional HIV retests postpartum provided only small incremental benefits (1 to 3 percentage point increase in HIV infections averted), and provision of one additional test at 14 weeks postpartum‐dominated scenarios in which this test was offered at six, nine, or both six and nine months postpartum. HIV retesting in late ANC and every three months postpartum was the most effective and most costly strategy, exceeding the cost‐effectiveness thresholds with ICERs of $7639 per DALY averted in Kenya and $11 985 in South Africa.

**Table 3:** Infant HIV infections, deaths, disability‐adjusted life years (DALYs) averted and cost‐effectiveness of maternal HIV retesting scenarios vs. base case scenario

Retesting scenario	Infant infections	Total infections averted	% Infections averted^d^	Infant deaths	Total cost	Incremental cost	Total DALYs	Incremental DALYs averted	ICER^e^
KENYA									
1. No retesting	13 484	–	–	61 651	$60 887 865	–	1 405 727	–	–
2. Late ANC^a^, ^b^	10 911	2573	19%	61 226	$63 295 344	$2 407 479	1 391 202	14 525	$166
5. Late ANC + 9 months	10 785	2699	20%	61 205	$66 901 930	–	1 390 493	–	*Dom*
3. Late ANC + 14 weeks	10 656	2828	21%	61 184	$67 067 821	$3 772 477	1 389 764	1438	$2,623
4. Late ANC + 6 months	10 766	2717	20%	61 202	$67 253 849	–	1 390 387	–	*Dom*
6. Late ANC + 6 months + 9 months	10 690	2793	21%	61 190	$70 505 634	–	1 389 957	–	*Dom*
7. Late ANC + every 3 months^c^	10 502	2982	22%	61 158	$73 717 459	$6 649 639	1 388 893	871	$7639
SOUTH AFRICA									
1. No retesting	27 038	–	–	41 077	$144 227 239	–	906 680	–	–
2. Late ANC^a^, ^b^	23 838	3200	12%	40 564	$149 363 142	$5 135 903	888 889	17 792	$289
5. Late ANC + 9 months	23 747	3290	12%	40 550	$153 742 500	––	888 387	–	*Dom*
3. Late ANC + 14 weeks	23 616	3421	13%	40 529	$154 454 205	$5 091 063	887 657	1232	$4134
4. Late ANC + 6 months	23 703	3334	12%	40 543	$155 719 914	–	888 141	–	*Dom*
6. Late ANC + 6 months + 9 months	23 650	3387	13%	40 534	$159 530 970	–	887 847	–	*Dom*
7. Late ANC + every 3 months^c^	23 475	3562	13%	40 506	$163 855 053	$9 400 848	886 873	784	$11 985
COLOMBIA									
1. No retesting	138	–	–	4530	$4 658 472	–	89 810	–	‐
2. Late ANC^a^, ^b^	115	23	17%	4525	$7 228 725	$2 570 253	89 648	162	$15 859
3. Late ANC + 14 weeks	111	28	20%	4524	$9 284 324	$2 055 599	89 615	34	$61 080
5. Late ANC + 9 months	112	26	19%	4524	$9 324 935	–	89 626	–	*Dom*
4. Late ANC + 6 months	113	26	19%	4524	$9 373 546	–	89 629	–	*Dom*
6. Late ANC + 6 months + 9 months	111	27	20%	4524	$11 433 846	–	89 616	–	*Dom*
7. Late ANC + every 3 months^c^	107	31	23%	4523	$13 440 069	$4 155 745	89 590	24	$170 418
UKRAINE									
1. No retesting	71	–	–	3101	$8 470 318	–	57 636	–	–
2. Late ANC^a^, ^b^	67	4	6%	3100	$9 960 198	$1 489 880	57 615	22	$69 107
3. Late ANC + 14 weeks	66	5	7%	3100	$11 345 338	$1 385 140	57 610	4	$316 654
5. Late ANC + 9 months	66	5	6%	3100	$11 372 961	–	57 612	–	*Dom*
4. Late ANC + 6 months	67	5	6%	3100	$11 397 314	–	57 612	–	*Dom*
6. Late ANC + 6 months + 9 months	66	5	7%	3100	$12 799 222	–	57 611	–	*Dom*
7. Late ANC + every 3 months^c^	66	5	8%	3100	$14 168 275	$2 822 937	57 608	3	$1 087 274

ANC, antenatal care; PP, postpartum; DALY, disability‐adjusted life‐year; Dom, dominated (more costly and less effective than dominating scenario).

^a^ Late ANC is between 36 to 39 weeks of gestation

^b^ Testing offered in late ANC, or at delivery if not performed at late ANC, or at six week MCH visit if not performed at delivery or late ANC

^c^ Approximately every three months PP includes retesting at six weeks, 14 weeks, six months and nine months PP

^d^ % infections averted is calculated by dividing total infections averted by the total number of infant infections that occur under Scenario 1 (no retesting)

^e^ ICER, incremental cost‐effectiveness ratio, calculated as incremental costs (in 2017 US$) divided by DALYs averted compared with the next least‐costly scenario with dominated and weakly dominated scenarios removed.

In Colombia, HIV retesting in late ANC with early postpartum catch‐up averted a similar proportion of HIV infections (17%) as in Kenya and South Africa (19% and 12% respectively), whereas this strategy averted fewer infections in Ukraine (6%). However, due to the low HIV burden, the absolute number of infections prevented was small in both countries (23 in Colombia and 4 in Ukraine). Adding additional HIV retests followed a similar pattern to that of Kenya and South Africa, providing marginal incremental benefits but with substantially higher costs. All HIV retesting scenarios in Colombia and Ukraine were either dominated or exceeded the cost‐effectiveness thresholds.

When restricting our results to women eligible for retesting (those without known HIV infection at the start of pregnancy), retesting in late ANC with early postpartum catch‐up testing averted 23% of infections in Kenya, 19% in South Africa, 20% in Colombia and 24% in Ukraine. Consistent with our main results, additional postpartum retests increased the percent of maximum potential infections averted but with diminishing returns.

### 3.1 Scenario implementation and uncertainty analyses

**Figure 2:**
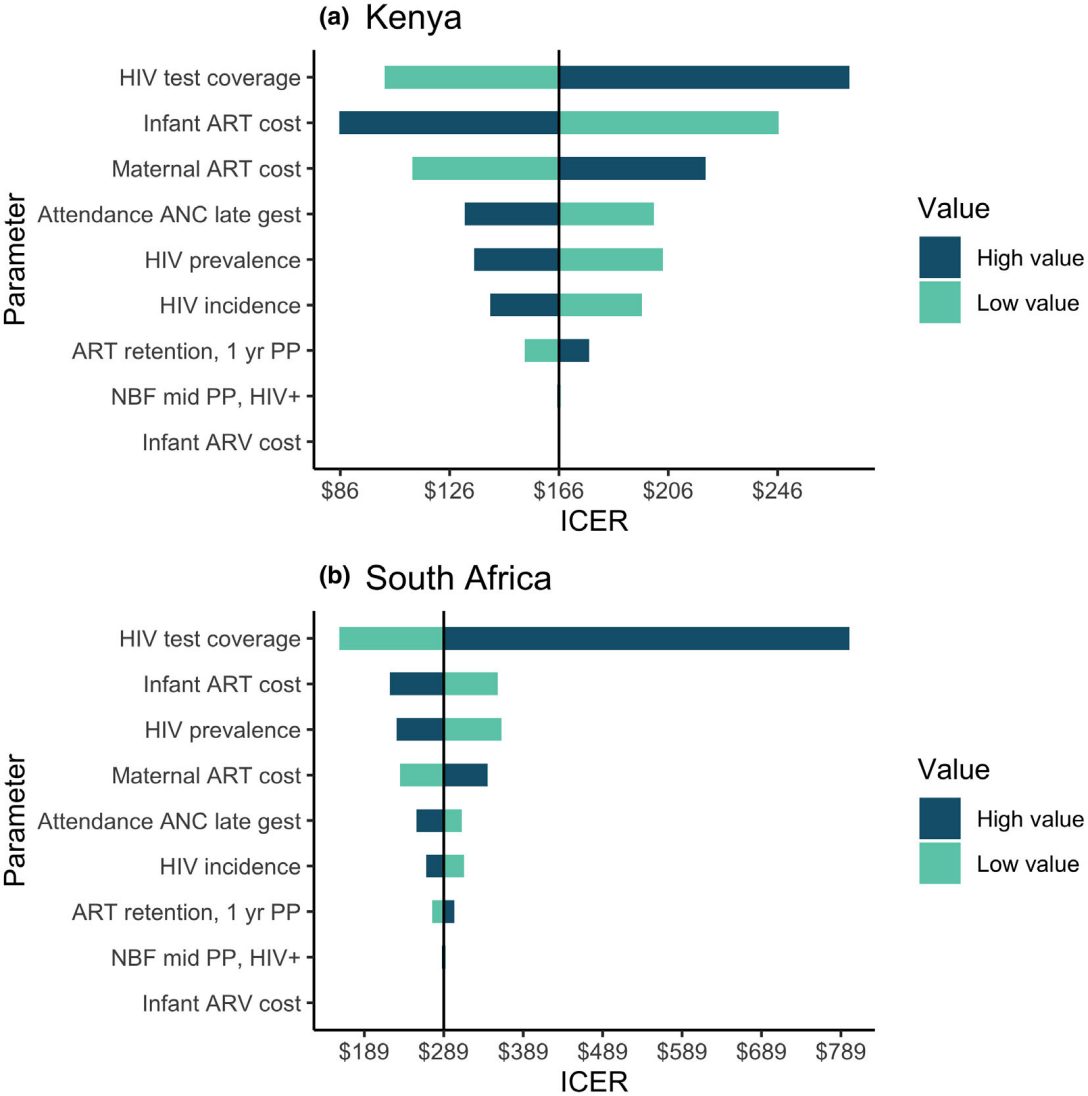
One‐way parameter uncertainty analyses in (a) Kenya and (b) South Africa under retesting Scenario 2 (retesting in late ANC/delivery/six weeks postpartum). Models included 20% relative increases and decreases in individual parameters, bounded by 0 and 1 for probabilities. ANC, antenatal care visit; PP, postpartum; ART, antiretroviral therapy; NBF, not breastfeeding; ARV, antiretroviral prophylaxis; HIV+, HIV positive; ICER: incremental cost‐effectiveness ratio, calculated as incremental costs (in 2017 US$) per disability‐adjusted life year averted. HIV incidence refers to both incidence between onset of pregnancy and first antenatal care (ANC) visit, and incidence between first ANC and delivery, with both parameters varied by 20% for this analysis. HIV test coverage is the product of test acceptance and test kit being in‐stock, with the composite parameter (test coverage) varied by 20% for this analysis.

**Figure 3:**
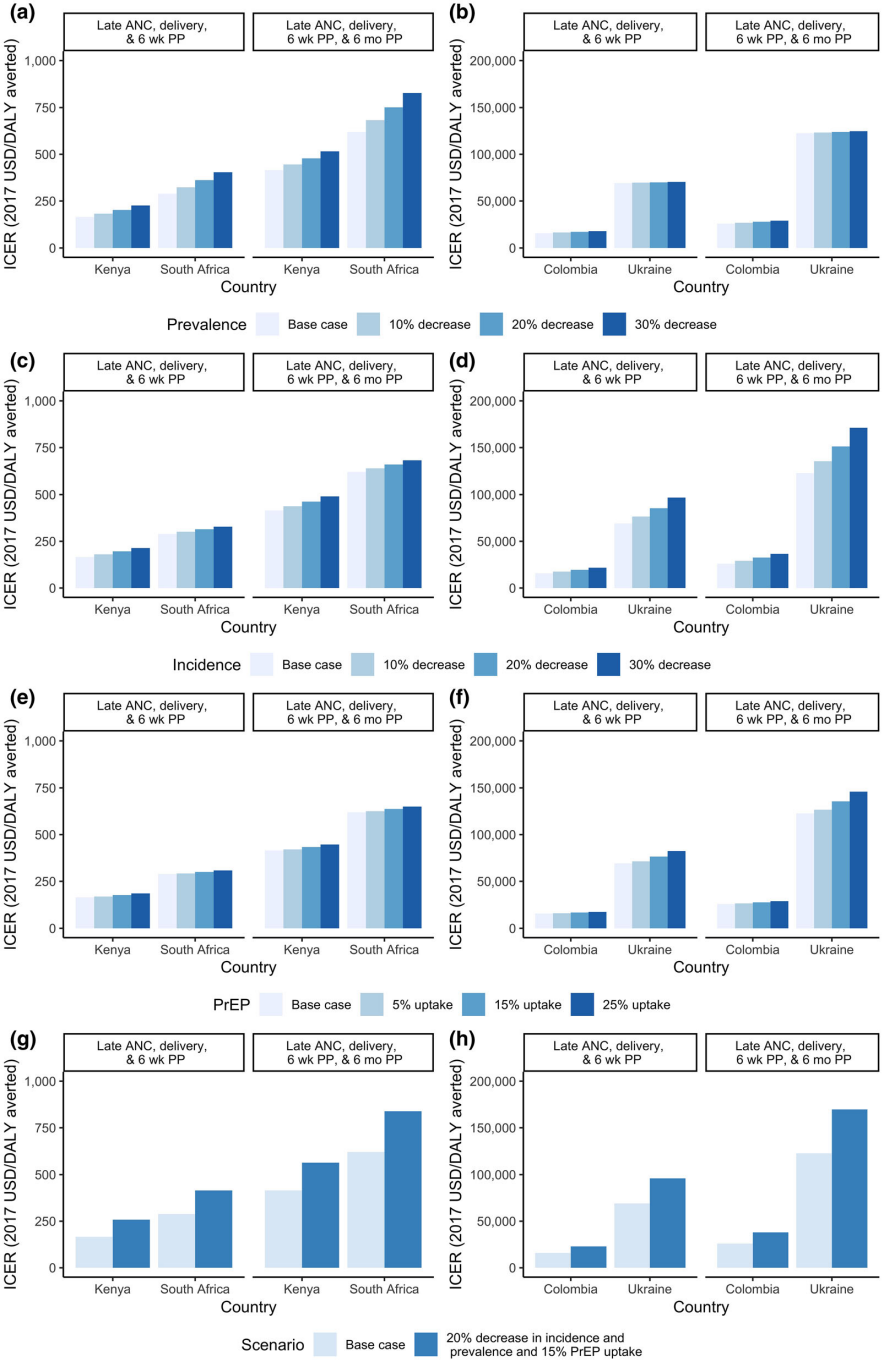
Scenario implementation analysis of maternal HIV retesting. (a) Decreasing HIV prevalence in Kenya and South Africa, (b) Decreasing HIV prevalence in Colombia and Ukraine, (c) Decreasing HIV incidence in Kenya and South Africa, (d) Decreasing HIV incidence in Colombia and Ukraine, (e) Increasing PrEP use in Kenya and South Africa, (f) Increasing PrEP use in Colombia and Ukraine, (g) Decreasing HIV prevalence and incidence, increasing PrEP use in Kenya and South Africa, (h) Decreasing HIV prevalence and incidence, increasing PrEP use in Colombia and Ukraine. ANC, antenatal care; PrEP, pre‐exposure prophylaxis; ICER, incremental cost‐effectiveness ratio; DALY, disability‐adjusted life years. Maternal HIV retesting Scenarios 2 (retesting in late ANC/delivery/six weeks postpartum) and 3 (retesting in late ANC/delivery/six weeks postpartum and at six months postpartum) modelled. ICERs are calculated with respect to Scenario 1 (no retesting).

In uncertainty analyses, ICERs for HIV retesting in late ANC with catch‐up retesting through six weeks postpartum ranged from $102 to $272/DALY averted in Kenya and from $156 to $799 for South Africa (Figure 2). Changes in HIV test coverage, followed by infant ART cost, had the largest impact on ICERs. In all countries, increases in PrEP use, decreases in HIV incidence, and decreases in HIV prevalence resulted in larger ICERs (Figure 3a‐h) and fewer HIV infections averted (Figure S1). All scenario analyses of retesting in late ANC in Kenya and South Africa remained below the cost‐effectiveness thresholds. Maternal retesting impact on MTCT was lowest when maternal incidence was reduced by 30%. Under an optimized care cascade, retesting in late ANC (with catch‐up testing) and every three months postpartum averted 19% to 22% of infections in South Africa and Kenya (Appendix S6).

## 4 DISCUSSION

We evaluated the health and economic impact of maternal HIV retesting at varying frequencies and intervals in four countries representing a range of HIV prevalence. In the intermediate‐ and high‐prevalence settings of Kenya and South Africa, our results indicate retesting in late ANC with catch‐up testing through early postpartum should be prioritized to capture as many maternal seroconversions occurring during pregnancy as possible. In such settings, retesting later in the postpartum period can capture maternal infections acquired after delivery but should only be considered in addition to retesting earlier in pregnancy/postpartum. As retesting in late ANC with catch‐up testing and quarterly postpartum (Scenario 7) most closely aligns with current South African guidelines [17], reducing the frequency of postpartum retesting may be effective in optimizing maternal HIV retesting with limited resources. In the low prevalence settings of Columbia and Ukraine, all HIV retesting strategies were dominated or greatly exceeded cost‐effectiveness thresholds. Retesting in late ANC with catch‐up testing prevented the largest number of infant HIV infections, but the absolute number was small (<25 infections annually).

Our model results also highlight limits on retesting as a strategy for EMTCT. While one‐third of infant infections are attributable to incident maternal infections [4], even with frequent testing throughout pregnancy and postpartum the proportion of infant infections averted due to retesting was only 27% in Kenya and 21% in South Africa after excluding MTCT attributed to women diagnosed with HIV prior to pregnancy. Optimizing MCH attendance and HIV care cascade parameters had little impact on these results. Antibody‐based testing has low sensitivity during acute infection, when transmission risk is highest, making incident infections more difficult to detect. Our results thus suggest that achieving EMTCT will require additional interventions for prevention, such as PrEP, partner testing or diagnostics that facilitate earlier detection of incident infections.

In Colombia and Ukraine, where postnatal risk of maternal HIV acquisition is low, as is subsequent MTCT risk due to low rates of breastfeeding, the impact of postpartum retesting is similar to antenatal retesting. Decisions on implementation of maternal retesting will depend on national priorities (such as EMTCT), programmatic goals and targets, and available resources, and countries with low HIV prevalence may consider retesting women at higher risk of HIV, including key populations (e.g. women who inject drugs, female sex workers) and those in serodiscordant relationships. Risk‐based screening tools, such as the one developed for pregnant and postpartum Kenyan women [39], may help identify such women during pregnancy or postpartum in both high and low prevalence settings.

Although our findings suggest maternal retesting is cost‐effective in countries with high maternal HIV prevalence, there are several factors that need to be considered when implementing this strategy. First, country guidelines need to provide clear criteria on retesting eligibility and timing [10]. Countries should consider provider capacity to offer retesting given their workload and ensure monitoring and evaluation activities both document testing history and measure impacts of retesting. Providers who offer testing will need training to deliver post‐test counselling messages that address HIV risk during pregnancy and breastfeeding and the need for future retesting, in addition to reinforcing HIV prevention messages. Emphasizing the importance of maternal retesting to prevent MTCT may also help providers prioritize retesting during busy ANC or postnatal care/infant immunization visits, including conveying elevated risks of HIV acquisition during pregnancy and breastfeeding and subsequent MTCT. Finally, test kit procurement and supply‐chain management must be optimized to ensure test kit availability at facilities and that maternal retesting does not hinder other HIV testing efforts.

We found additional postpartum retesting incurs high costs per DALY averted, thus countries adopting this approach will need innovative and differentiated retesting models to optimize resource use [2]. Because HIV test coverage at first ANC visit is high in most countries, alternative delivery approaches to retesting may increase coverage among women who only attend one ANC visit, deliver at home, and/or do not return for MCH visits for postnatal care/infant immunizations. The WHO recommends self‐testing as an additional testing approach [40]; however, to date few programmes have introduced self‐tests for maternal retesting. While self‐tests are unlikely to replace clinic‐based rapid tests, they may play a complementary role in PMTCT programmes to increase access and acceptability of retesting and should be evaluated for maternal retesting in future studies. In the context of the COVID‐19 response, due to declining and delayed facility visits in many settings, HIV self‐tests are increasingly being considered as a way to provide maternal catch‐up testing and retesting for programmes and should be considered and evaluated.

Our analysis has several strengths. We model delivery of HIV retesting at routine ANC and MCH visits, mitigating the costs incurred by retesting. A similar strategy could be adopted for HIV testing in conjunction with routine paediatric vaccination past the MCH visits included in our model. We assessed the impact of additional tests during the postnatal period, which is included in comparatively fewer national guidelines than retesting during late pregnancy/delivery [10]. Furthermore, we included countries with different HIV burden and geographic locations. While countries modelled are currently not implementing PrEP for pregnant and postpartum women at the national level, we assessed the impact of future PrEP scale‐up on retesting strategies in sensitivity analyses. We also modelled reductions in maternal HIV incidence that could result from other HIV prevention interventions that may benefit pregnant/lactating women (i.e. condom use, risk reduction, partner HIV testing) [40]. Our conclusions were robust to varying assumptions about HIV prevalence, incidence and PrEP scale‐up.

Our analysis also has several limitations. Some parameter values were assumed due to lack of empiric data; however, our estimates of MTCT rates were consistent with published estimates. While we modelled paediatric ART costs through 20 years, we modelled maternal ART costs only through 12 months postpartum, as maternal outcomes were not the focus of our model and we assume MTCT ceases after 12 months. We did not model any changes in infant ARV and paediatric ART costs over this period, but our results were robust to changes in costs in sensitivity analyses. HIV diagnosis and linkage to care improves maternal health outcomes and may also prevent horizontal transmissions, neither of which are not captured by our model. While our intent was to model retesting in late pregnancy, tests in late ANC capture both incident maternal infections and established infections not detected at first ANC due to late presentation (after the gestational age of first ANC modelled) or absence of testing at first ANC. Furthermore, our model only allows for testing at two antenatal time points, with parameterization corresponding to re‐testing in the third trimester in South Africa and Kenya, and in the second trimester in Colombia and Ukraine. As our model did not stratify by demographic or HIV risk factors, our results may not represent the impact of maternal retesting in concentrated epidemics within a country, nor of retesting prioritized for key populations. Our choice of countries was constrained by data availability and may not be generalizable to other settings where key parameters differ substantially from those modelled. We did not explicitly model time since maternal ART initiation and therefore may not have captured more complex temporal patterns in maternal ART use. Our model assumed all HIV‐infected infants were equally likely to receive ART regardless of whether their mother’s HIV‐positive status was known or unknown. As early diagnosis and treatment will avert DALYs even if transmission is not prevented, our results are conservative in this regard. Our results are presented as point estimates without accompanying uncertainty. As several of our model parameters were based on expert elicitation or assumptions, and we expect many are correlated, we felt valid parameterization of a formal probabilistic sensitivity analysis was out of reach. Finally, the cost‐effectiveness thresholds used in this analysis are approximate and may not accurately reflect country‐specific willingness‐to‐pay under budget constraints. Affordability constraints may prevent some countries from implementing interventions below these thresholds. Alternatively, in efforts to achieve EMTCT, programmes may consider incurring high costs to implement retesting strategies above the cost‐effectiveness thresholds used in this analysis.

## 5 CONCLUSIONS

We found retesting women in late ANC followed by catch‐up testing though six weeks postpartum is a cost‐effective approach to PMTCT in Kenya and South Africa and should be implemented by PMTCT programmes. Universal maternal retesting was not cost‐effective in Colombia and Ukraine; these counties may consider retesting if resources are available as retesting does contribute to EMTCT efforts, which would align with their current national retesting guidance. Additional empiric data on implementing maternal retesting guidelines is needed to refine model inputs, to evaluate retesting strategies for key populations at high HIV risk, and to revise retesting guidelines as incidence declines in high‐burden countries.

## COMPETING INTEREST

The authors declare they have no competing interests.

## AUTHORS’ CONTRIBUTIONS

JM, DAR, PR, RB, SE, MSJ, RB, CJ and ALD designed the study and JM, DAR, PR, BG, MBM, AB, AV, AS, GG, SF, SE, CJ and ALD collected data to populate the model. JM, DAR, PR, AD, MSJ, CJ and ALD contributed to the literature search. JM, DAR, PR, MS and ALD were responsible for constructing the model and analysing model results. All authors contributed to writing the manuscript and interpreting the results.

## Supporting information


**Figure S1.** Scenario implementation analysis of maternal HIV retesting on number of infant HIV infections averted. **(a)** Decreasing HIV prevalence in Kenya and South Africa, **(b)** Decreasing HIV prevalence in Colombia and Ukraine, **(c)** Decreasing HIV incidence in Kenya and South Africa, **(d)** Decreasing HIV incidence in Colombia and Ukraine, **(e)** Increasing PrEP use in Kenya and South Africa, **(f)** Increasing PrEP use in Colombia and Ukraine, **(g)** Decreasing HIV prevalence and incidence, increasing PrEP use in Kenya and South Africa, **(h)** Decreasing HIV prevalence and incidence, increasing PrEP use in Colombia and Ukraine. ANC, antenatal care; PrEP, pre‐exposure prophylaxis; Maternal HIV retesting Scenarios 2 (retesting in late ANC/delivery/six weeks postpartum) and 3 (retesting in late ANC/delivery/six weeks postpartum and at 6 months postpartum) modelled.

**Appendix S1.** Model hyperlink: https://github.com/dallenroberts/Maternal‐testing

**Appendix S2.** Model equations.

**Appendix S3.** Model parameters

**Appendix S4.** Comparison of model vs published estiamtes of maternal to child transmission

**Appendix S5.** Microcosting methods

**Appendix S6.** Infections averted under optimized MCH attendance and HIV care cascade

## References

[1] Elimination of mother‐to‐child transmission. UNICEF; 2019. [accessed 2019 Apr 8]. Available from: https://data.unicef.org/topic/hivaids/emtct/

[2] Global AIDS update 2019 — Communities at the centre. Geneva: UNAIDS Joint United Nations Programme on HIV/AIDS. 2019.

[3] Johnson LF , Stinson K , Newell ML , Bland RM , Moultrie H , Davies MA , et al. The contribution of maternal HIV seroconversion during late pregnancy and breastfeeding to mother‐to‐child transmission of HIV. J Acquir Immune Defic Syndr. 2012;59:417–25. 22193774 10.1097/QAI.0b013e3182432f27 PMC3378499

[4] UNAIDS . Start free stay free AIDS free 2019 report. Joint United Nations Programme on HIV/AIDS (UNAIDS).

[5] Drake AL , Wagner A , Richardson B , John‐Stewart G . Incident HIV during pregnancy and postpartum and risk of mother‐to‐child HIV transmission: a systematic review and meta‐analysis. PLoS Medicine. 2014;11:e1001608. 24586123 10.1371/journal.pmed.1001608 PMC3934828

[6] Fast Track: ending the AIDS epidemic by 2030 [press release]. Geneva: Joint United Nations Programme on HIV/AIDS (UNAIDS); 2014.

[7] Ishikawa N , Dalal S , Johnson C , Hogan DR , Shimbo T , Shaffer N , et al. Should HIV testing for all pregnant women continue? Cost‐effectiveness of universal antenatal testing compared to focused approaches across high to very low HIV prevalence settings. J Int AIDS Soc. 2016;19:21212. 27978939 10.7448/IAS.19.1.21212 PMC5159683

[8] WHO HIV/AIDS Programme . Antiretroviral drugs for treating pregnant women and preventing HIV infection in infants: towards universal access towards universal access. Recommendations for a public health approach. 2006.

[9] WHO . Consolidated guidelines on HIV testing services. Geneva, Switzerland: WHO; 2015.

[10] Drake AL , Thomson KA , Quinn C , Newman Owiredu M , Nuwagira IB , Chitembo L , et al. Retest and treat: a review of national HIV retesting guidelines to inform elimination of mother‐to‐child HIV transmission (EMTCT) efforts. J Int AIDS Society. 2019;22:e25271. 10.1002/jia2.25271 PMC6452920 30958644

[11] Joshi S , Kulkarni V , Gangakhedkar R , Mahajan U , Sharma S , Shirole D , et al. Cost‐effectiveness of a repeat HIV test in pregnancy in India. BMJ Open. 2015;5:e006718. 10.1136/bmjopen-2014-006718 PMC4466614 26068507

[12] Kim LH , Cohan DL , Sparks TN , Pilliod RA , Arinaitwe E , Caughey AB . The cost‐effectiveness of repeat HIV testing during pregnancy in a resource‐limited setting. J Acquir Immune Defic Syndr. 2013;63:195–200. 23392461 10.1097/QAI.0b013e3182895565 PMC3653987

[13] Soorapanth S , Sansom S , Bulterys M , Besser M , Theron G , Fowler MG . Cost‐effectiveness of HIV rescreening during late pregnancy to prevent mother‐to‐child HIV transmission in South Africa and other resource‐limited settings. J Acquir Immune Defic Syndr. 2006;42:213–21. 16639346 10.1097/01.qai.0000214812.72916.bc

[14] Kinuthia J , Richardson BA , Drake AL , Matemo D , Unger JA , McClelland RS , et al. Sexual behavior and vaginal practices during pregnancy and postpartum: implications for HIV prevention strategies. J Acquir Immune Defic Syndr. 2017;74(2):142–9. 27828872 10.1097/QAI.0000000000001225 PMC5357239

[15] Thomson KA , Hughes J , Baeten JM , John‐Stewart G , Celum C , Cohen CR , et al. Increased risk of HIV acquisition among women throughout pregnancy and during the postpartum period: a prospective per‐coital‐act analysis among women with HIV‐infected partners. J Infect Dis. 2018;218(1):16–25. 29514254 10.1093/infdis/jiy113 PMC5989601

[16] UNAIDS . Resources and financing. [accessed 2019 Apr 8]. Available from: https://www.unaids.org/en/topic/resources

[17] National Department of Health . National HIV Testing Services: Policy. Republic of South Africa: Department of Health; 2016. [accessed 2018 Apr 8]. Available from: https://sahivsoc.org/Files/HTS%20Policy%2028%20July%20final%20copy.pdf

[18] Minstry of Health, National AIDS & STI Control Programme . Guidelines on Use of Antiretroviral Drugs for Treating and Preventing HIV infection in Kenya. Nairobi, Kenya: Ministry of Health Kenya, National AIDS & STI Control Program; 2016.

[19] Bozicevic ID , Z. Report on pre‐validation of elimination of mother‐to‐child transmission of HIV in Ukraine. December 2018.

[20] Guía de práctica clínica (GPC) basada en la evidencia científica para la atención de la infección por VIH/Sida en adolescentes (con 13 años o más de edad) y adultos. Guía para profesionales de la salud GPC‐2014‐39. Libert y Orden. In: Social MdSyP, editor. Bogata, Colombia. December 2014.

[21] WHO . Consolidated guidelines on the use of antiretroviral drugs for treating and preventing HIV infection. 2nd edition. 2016. [accessed 2019 Apr 8]. Available from: http://apps.who.int/iris/bitstream/handle/10665/208825/9789241549684_eng.pdf

[22] Haas AD , Tenthani L , Msukwa MT , Tal K , Jahn A , Gadabu OJ , et al. Retention in care during the first 3 years of antiretroviral therapy for women in Malawi's option B+ programme: an observational cohort study. Lancet HIV. 2016;3:e175–e182. 27036993 10.1016/S2352-3018(16)00008-4 PMC4904064

[23] McGrath CJ , Singa B , Langat A , Kinuthia J , Ronen K , Omolo D , et al. Non‐disclosure to male partners and incomplete PMTCT regimens associated with higher risk of mother‐to‐child HIV transmission: a national survey in Kenya. AIDS Care. 2018;30(6):765–73. 29130333 10.1080/09540121.2017.1400642 PMC5895526

[24] Johnson LF , May MT , Dorrington RE , Cornell M , Boulle A , Egger M , et al. Estimating the impact of antiretroviral treatment on adult mortality trends in South Africa: a mathematical modelling study. PLoS Medicine. 2017;14:e1002468. 29232366 10.1371/journal.pmed.1002468 PMC5726614

[25] Elimination of mother‐to‐child transmission of HIV and syphilis in the Americas. Update 2016. Washington, DC: PAHO; 2017.

[26] U.S. Bureau of Labor Statistics . CPI Inflation Calculator. [accessed 2019 Apr 8]. Available from: https://www.bls.gov/data/inflation_calculator.htm

[27] Vassall A , Sweeney S , Kahn J , Gomez G , Bollinger L , Marseille E , et al. Reference Case for Estimating the Costs of Global Health Services and Interventions. 2017. [accessed 2019 Apr 8]. Available from: https://ghcosting.org/pages/standards/reference_case

[28] Department of Information Evidence and Research . WHO methods and data sources for global burden of disease estimates. Geneva, 2018. [accessed 2019 Apr 8]. Available from: http://www.who.int/healthinfo/global_burden_disease/GlobalDALY_method_2000_2016.pdf

[29] World Health Organization . Country Life Tables. 2016. [accessed 2019 Apr 8]. Available from: http://apps.who.int/gho/data/?theme=main&vid=60850

[30] Newell ML , Coovadia H , Cortina‐Borja M , Rollins N , Gaillard P , Dabis F , et al. Mortality of infected and uninfected infants born to HIV‐infected mothers in Africa: a pooled analysis. Lancet. 2004;364(9441):1236–43. 15464184 10.1016/S0140-6736(04)17140-7

[31] Drummond MF , Sculpher MJ , Claxton K , Stoddart GL , Torrence GW . Methods for the economic evaluation of health care programmes, 4thd edn. Oxford, England: Oxford University Press; 2015.

[32] Phillips AN , Cambiano V , Nakagawa F , Revill P , Jordan MR , Hallett TB , et al. Cost‐effectiveness of public‐health policy options in the presence of pretreatment NNRTI drug resistance in sub‐Saharan Africa: a modelling study. Lancet HIV. 2018;5(3):e146–e154. 29174084 10.1016/S2352-3018(17)30190-X PMC5843989

[33] Woods B , Revill P , Sculpher M , Claxton K . Country‐level cost‐effectiveness thresholds: initial estimates and the need for further research. Value Health. 2016;19(8):929–35. 27987642 10.1016/j.jval.2016.02.017 PMC5193154

[34] Meyer‐Rath G , van Rensburg C , Larson B , Jamieson L , Rosen S . Revealed willingness‐to‐pay versus standard cost‐effectiveness thresholds: evidence from the South African HIV investment case. PLoS One. 2017;12:e0186496. 29073167 10.1371/journal.pone.0186496 PMC5658054

[35] Phillips AN , Cambiano V , Johnson L , Nakagawa F , Homan R , Meyer‐Rath G , et al. Potential impact and cost‐effectiveness of condomless‐sex‐concentrated PrEP in KwaZulu‐Natal accounting for drug resistance. J Infect Dis. 2019:jiz667. 10.1093/infdis/jiz667 31851759 PMC8064039

[36] Topachevskyi O , Piniazhko O , Lebega O , Oleshchuk O . Estimation of supply side cost effectiveness threshold in Ukraine: perspective use in health care decision‐making. Value in Health. 2018;21:S100.

[37] Guidelines for the economic evaluation of healthcare technologies in Colombia: technical support documents. Bogota, DC: IETS; 2014.

[38] Husereau D , Drummond M , Petrou S , Carswell C , Moher D , Greenberg D , et al. Consolidated health economic evaluation reporting standards (CHEERS)–explanation and elaboration: a report of the ISPOR health economic evaluation publication guidelines good reporting practices task force. Value Health. 2013;16(2):231–50. 23538175 10.1016/j.jval.2013.02.002

[39] Pintye J , Drake AL , Kinuthia J , Unger JA , Matemo D , Heffron RA , et al. A risk assessment tool for identifying pregnant and postpartum women who may benefit from preexposure prophylaxis. Clin Infect Dis. 2017;64(6):751–8. 28034882 10.1093/cid/ciw850 PMC6075205

[40] WHO Technical Brief . Preventing HIV during pregnancy and breastfeeding in the context of PrEP. 2017. [accessed 2019 Apr 8]. Available from: http://www.who.int/hiv/pub/toolkits/prep‐preventing‐hiv‐during‐pregnancy/en/

[41] GAVI . Country hub: Kenya. [accessed 2019 Apr 8]. Available from: https://www.gavi.org/country/kenya/

[42] World Health Organization . Syphilis estimation tool. [accessed 2019 Apr 8]. Available from: https://www.who.int/reproductivehealth/topics/rtis/syphilis/measurement_tool/en/

[43] UNAIDS Country Factsheets. Kenya. 2018. [accessed 2020 Jan 27]. Available from: https://www.unaids.org/en/regionscountries/countries/kenya

[44] Woldesenbet SA , Kufa T , Lombard C , Manda S , Ayalew K , Cheyip M , et al. The 2017 National antenatal sentinel HIV survey. National Department of Health: South Africa; 2019.

[45] UNAIDS . Prevalence of HIV (Ages 15‐49) ‐ South America. [accessed 2019 Apr 8]. Available from: https://www.indexmundi.com/facts/indicators/SH.DYN.AIDS.ZS/map/south‐america

[46] UNAIDS Country Factsheet. Ukraine. 2018. [accessed 2020 Jan 14]. Available from: https://www.unaids.org/en/regionscountries/countries/ukraine

[47] Kinuthia J , Drake AL , Matemo D , Richardson BA , Zeh C , Osborn L , et al. HIV acquisition during pregnancy and postpartum is associated with genital infections and partnership characteristics. AIDS. 2015;29:2025–33. 26352880 10.1097/QAD.0000000000000793 PMC4692052

[48] UNAIDS . Data 2018. [accessed 2019 Apr 8]. Available from: https://www.unaids.org/sites/default/files/media_asset/unaids‐data‐2018_en.pdf

[49] Kohler PK , Okanda J , Kinuthia J , Mills LA , Olilo G , Odhiambo F , et al. Community‐based evaluation of PMTCT uptake in Nyanza Province. Kenya. PloS one. 2014;9:e110110. 25360758 10.1371/journal.pone.0110110 PMC4215877

[50] Myer L , Phillips T , Manuelli V , McIntyre J , Bekker LG , Abrams EJ . Evolution of antiretroviral therapy services for HIV‐infected pregnant women in Cape Town, South Africa. J Acquir Immune Defic Syndr. 2015;69(2):e57–e65. 25723138 10.1097/QAI.0000000000000584 PMC4550573

[51] Sirengo M , Muthoni L , Kellogg TA , Kim AA , Katana A , Mwanyumba S , et al. Mother‐to‐child transmission of HIV in Kenya: results from a nationally representative study. J Acquir immune Defic syndr. 1999;2014 66 Suppl 1:S66–74. 10.1097/QAI.0000000000000115 PMC4790087 24732822

[52] DHS Program . South Africa Demographic and Health Survey. 2016. [accessed 2019 Apr 8]. Available from: https://www.dhsprogram.com/publications/publication‐FR337‐DHS‐Final‐Reports.cfm

[53] WHO Prequalification of In Vitro Diagnostics Public Report. Product: Alere™ HIV Combo WHO reference number: PQDx 0243‐013‐00. September 2018, version 4.0. [accessed 2019 Apr 8]. Available from: https://www.who.int/diagnostics_laboratory/evaluations/pq‐list/hiv‐rdts/180913_amended_final_pqpr_0033_013_00_v6.pdf?ua=1

[54] Coovadia HM , Rollins NC , Bland RM , Little K , Coutsoudis A , Bennish ML , et al. Mother‐to‐child transmission of HIV‐1 infection during exclusive breastfeeding in the first 6 months of life: an intervention cohort study. Lancet. 2007;369:1107–16. 17398310 10.1016/S0140-6736(07)60283-9

[55] Duri K , Gumbo FZ , Kristiansen KI , Kurewa NE , Mapingure MP , Rusakaniko S , et al. Antenatal HIV‐1 RNA load and timing of mother to child transmission; a nested case‐control study in a resource poor setting. Virol J. 2010;7:176. 20678190 10.1186/1743-422X-7-176 PMC2919474

[56] Liang K , Gui X , Zhang YZ , Zhuang K , Meyers K , Ho DD . A case series of 104 women infected with HIV‐1 via blood transfusion postnatally: high rate of HIV‐1 transmission to infants through breast‐feeding. J Infect Dis. 2009;200:682–6. 19627245 10.1086/605123

[57] Marinda ET , Moulton LH , Humphrey JH , Hargrove JW , Ntozini R , Mutasa K , et al. In utero and intra‐partum HIV‐1 transmission and acute HIV‐1 infection during pregnancy: using the BED capture enzyme‐immunoassay as a surrogate marker for acute infection. Int J Epidemiol. 2011;40:945–54. 21471020 10.1093/ije/dyr055 PMC3156369

[58] Zijenah LS , Moulton LH , Iliff P , Nathoo K , Munjoma MW , Mutasa K , et al. Timing of mother‐to‐child transmission of HIV‐1 and infant mortality in the first 6 months of life in Harare, Zimbabwe. AIDS. 2004;18:273–80. 15075545 10.1097/00002030-200401230-00017

[59] Kenya National Bureau of Statistics, Kenya Ministry of Health, Kenya National AIDS Control Council, Kenya Medical Research Institute, and Kenya National Council for Population and Development . Kenya Demographic and Health Survey 2014. 2015.

[60] DHS Program . Ukraine Demographic and Health Survey. 2007. [accessed 2019 Apr 8]. Available from: https://dhsprogram.com/publications/publication‐FR210‐DHS‐Final‐Reports.cfm

[61] World Health Organization . WHO‐UNICEF Estimates of DTP1 Coverage. 2018 [cited 2019 May 9, accessed 2019 Apr 8]. Available from: http://apps.who.int/immunization_monitoring/globalsummary/timeseries/tswucoveragedtp1.html

[62] ETMI. PLUS . Estrategia Nacional para la Eliminación de la Transmisión Materno Infantil del VIH, la sífilis congénita, la hepatitis B y la enfermedad de Chagas. Comportamiento de la Transmisión Materno Infantil del VIH en Colombia. Medición de la Cohorte. 2017;. Dirección de Promoción y Prevención Grupo de Sexualidad, Derechos Sexuales y Derechos Reproductivos. Colombia 29 de mayo de 2019.

[63] Snippenburg W , Nellen F , Smit C , Wensing A , Godfried MH , Mudrikova T . Factors associated with time to achieve an undetectable HIV RNA viral load after start of antiretroviral treatment in HIV‐1‐infected pregnant women. J Virus Eradic. 2017;3:34–9. 10.1016/S2055-6640(20)30294-6 PMC5337419 28275456

[64] Brittain K , Mellins CA , Remien RH , Phillips TK , Zerbe A , Abrams EJ , et al. Impact of HIV‐status disclosure on HIV viral load in pregnant and postpartum women on antiretroviral therapy. J Acquir Immune Defic Syndr. 2019;81(4):379–86. 30939530 10.1097/QAI.0000000000002036 PMC6594888

[65] Jones KR , Lekhak N , Kaewluang N . Using mobile phones and short message service to deliver self‐management interventions for chronic conditions: a meta‐review. Worldviews Evid Based Nurs. 2014;11(2):81–8. 24597522 10.1111/wvn.12030

[66] West NS , Schwartz SR , Yende N , Schwartz SJ , Parmley L , Gadarowski MB , et al. Infant feeding by South African mothers living with HIV: implications for future training of health care workers and the need for consistent counseling. Int Breastfeed J. 2019;14:11. 30815026 10.1186/s13006-019-0205-1 PMC6376722

[67] Wringe A , Floyd S , Kazooba P , Mushati P , Baisley K , Urassa M , et al. Antiretroviral therapy uptake and coverage in four HIV community cohort studies in sub‐Saharan Africa. Trop Med Int Health. 2012;17(8):e38–48. 22943378 10.1111/j.1365-3156.2011.02925.x PMC3443383

[68] World Bank . Maternal mortality ratio modeled estimates, 2017. 2019. [accessed 2019 Apr 8]. Available from: https://data.worldbank.org/indicator/sh.sta.mmrt

[69] Institute for Health Metrics and Evaluation . Global burden of disease estimates. 2017. [accessed 2019 Apr 8]. Available from: http://ghdx.healthdata.org/gbd‐results‐tool

[70] Adebajo S , Eluwa G , Njab J , Oginni A , Ukwuije F , Ahonsi B , et al. Evaluating the effect of HIV prevention strategies on uptake of HIV counselling and testing among male most‐at‐risk‐populations in Nigeria; a cross‐sectional analysis. Sex Transm Infect. 2015;91(8):555–60. 25921019 10.1136/sextrans-2014-051659

[71] Larson BA , Bii M , Halim N , Rohr JK , Sugut W , Sawe F . Incremental treatmet costs for HIV‐infected women initiating antiretroviral therapy during pregnancy: A 24‐month micro‐costing cohort study for a maternal and child health clinic in Kenya. PLoS One. 2018;13:e0200199. 30096177 10.1371/journal.pone.0200199 PMC6086393

[72] Meyer‐Rath G , van Rensburg C , Chiu C , Leuner R , Jamieson L , Cohen S . The per‐patient costs of HIV services in South Africa: Systematic review and application in the South African HIV Investment Case. PLoS One. 2019;14:e0210497. 30807573 10.1371/journal.pone.0210497 PMC6391029

[73] The World Bank . Ukraine HIV Program Efficiency Study: Can Ukraine improve value for money in HIV service delivery?. Washington DC: 2013.

[74] Bautista‐Arredondo S , Sosa‐Rubí SG , Opuni M , Contreras‐Loya D , Kwan A , Chaumont C , et al. Costs along the service cascades for HIV testing and counselling and prevention of mother‐to‐child transmission. AIDS. 2013;30:2495–504. 10.1097/QAD.0000000000001208 PMC5051528 27753679

[75] Roberts DA , Barnabas RV , Abuna F , Lagat H , Kinuthia J , Pintye J , et al. The role of costing in the introduction and scale‐up of HIV pre‐exposure prophylaxis: evidence from integrating PrEP into routine maternal and child health and family planning clinics in western Kenya. J Int AIDS Soc. 2019;22:e25296. 31328443 10.1002/jia2.25296 PMC6643078

[76] Alistar SS , Owens DK , Brandeau ML . Effectiveness and cost effectiveness of oral pre‐exposure prophylaxis in a portfolio of prevention programs for injection drug users in mixed HIV epidemics. PLoS One. 2014;9:e86584. 24489747 10.1371/journal.pone.0086584 PMC3904940

[77] CDC, Kenya Ministry of Health . The cost of comprehensive HIV treatment in Kenya. 2013.

[78] UNAIDS Country Factsheets. South Africa. 2018. [accessed 2020 Jan 27]. Available from: https://www.unaids.org/en/regionscountries/countries/southafrica

