# Supplementary figures and images for: Optimizing HIV retesting during pregnancy and postpartum in four countries: a cost‐effectiveness analysis
Source: J Int AIDS Soc. 2021 Mar 31;24(4):e25686. doi: 10.1002/jia2.25686 (PMC8010369; doi:10.1002/jia2.25686)

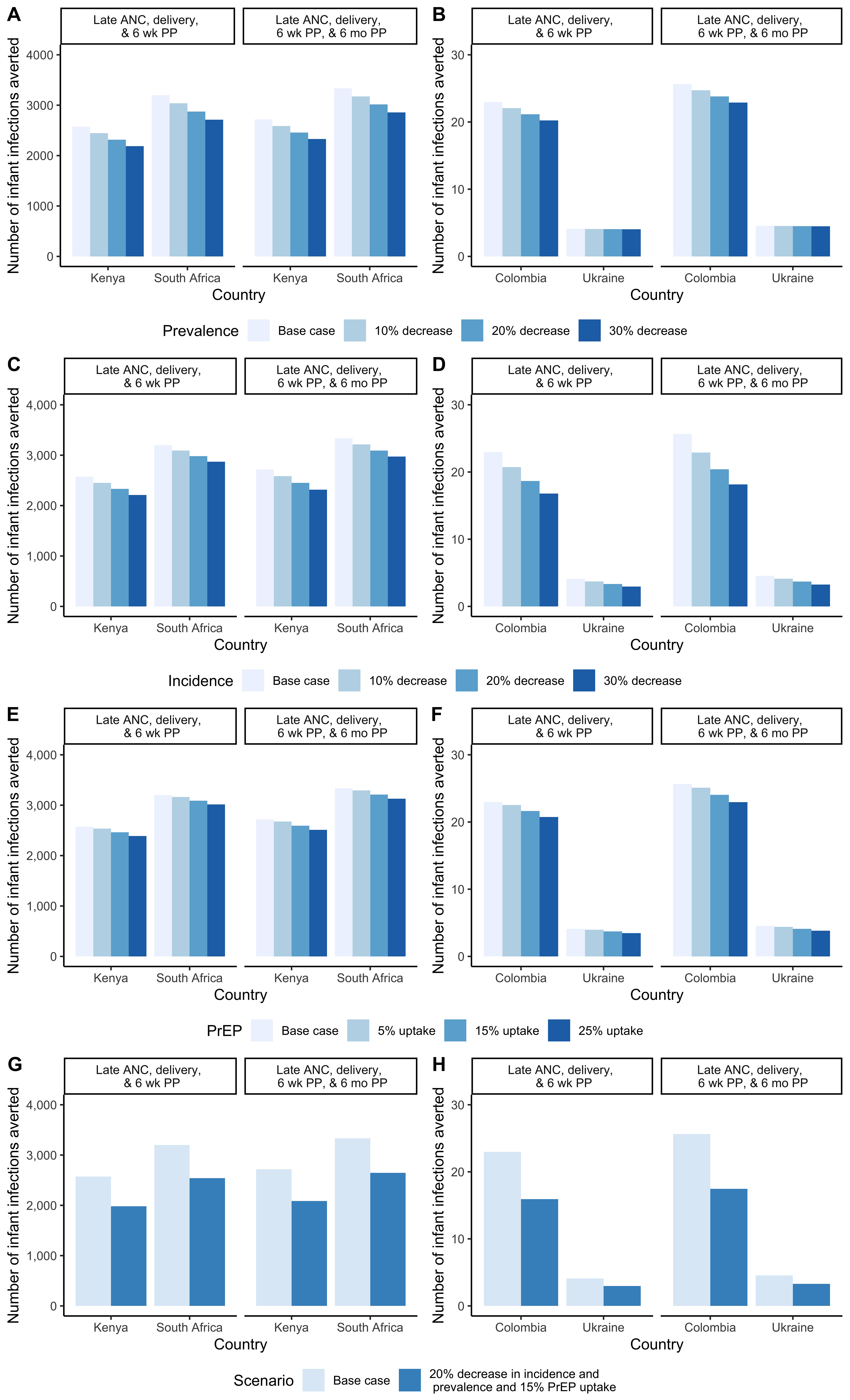

Supplement: Supplementary file 1 — Figure S1. Scenario implementation analysis of maternal HIV retesting on number of infant HIV infections averted. (a) Decreasing HIV prevalence in Kenya and South Africa, (b) Decreasing HIV prevalence in Colombia and Ukraine, (c) Decreasing HIV incidence in Kenya and South Africa, (d) Decreasing HIV incidence in Colombia and Ukraine, (e) Increasing PrEP use in Kenya and South Africa, (f) Increasing PrEP use in Colombia and Ukraine, (g) Decreasing HIV prevalence and incidence, increasing PrEP use in Kenya and South Africa, (h) Decreasing HIV prevalence and incidence, increasing PrEP use in Colombia and Ukraine. ANC, antenatal care; PrEP, pre‐exposure prophylaxis; Maternal HIV retesting Scenarios 2 (retesting in late ANC/delivery/six weeks postpartum) and 3 (retesting in late ANC/delivery/six weeks postpartum and at 6 months postpartum) modelled. [file JIA2-24-e25686-s002.tif]
