# Supplementary material for: Optimizing HIV retesting during pregnancy and postpartum in four countries: a cost‐effectiveness analysis
Source: J Int AIDS Soc. 2021 Mar 31;24(4):e25686. doi: 10.1002/jia2.25686 (PMC8010369; doi:10.1002/jia2.25686)
Supplement: Supplementary file 2 — Appendix S1. Model hyperlink: https://github.com/dallenroberts/Maternal‐testing [file JIA2-24-e25686-s005.docx]

Appendix 1:

Model hyperlink: <https://github.com/dallenroberts/Maternal-testing>
