# Supplementary material for: Optimizing HIV retesting during pregnancy and postpartum in four countries: a cost‐effectiveness analysis
Source: J Int AIDS Soc. 2021 Mar 31;24(4):e25686. doi: 10.1002/jia2.25686 (PMC8010369; doi:10.1002/jia2.25686)
Supplement: Supplementary file 3 — Appendix S2. Model equations. [file JIA2-24-e25686-s003.docx]

Appendix 2: Model equations

## 1 Maternal state transitions


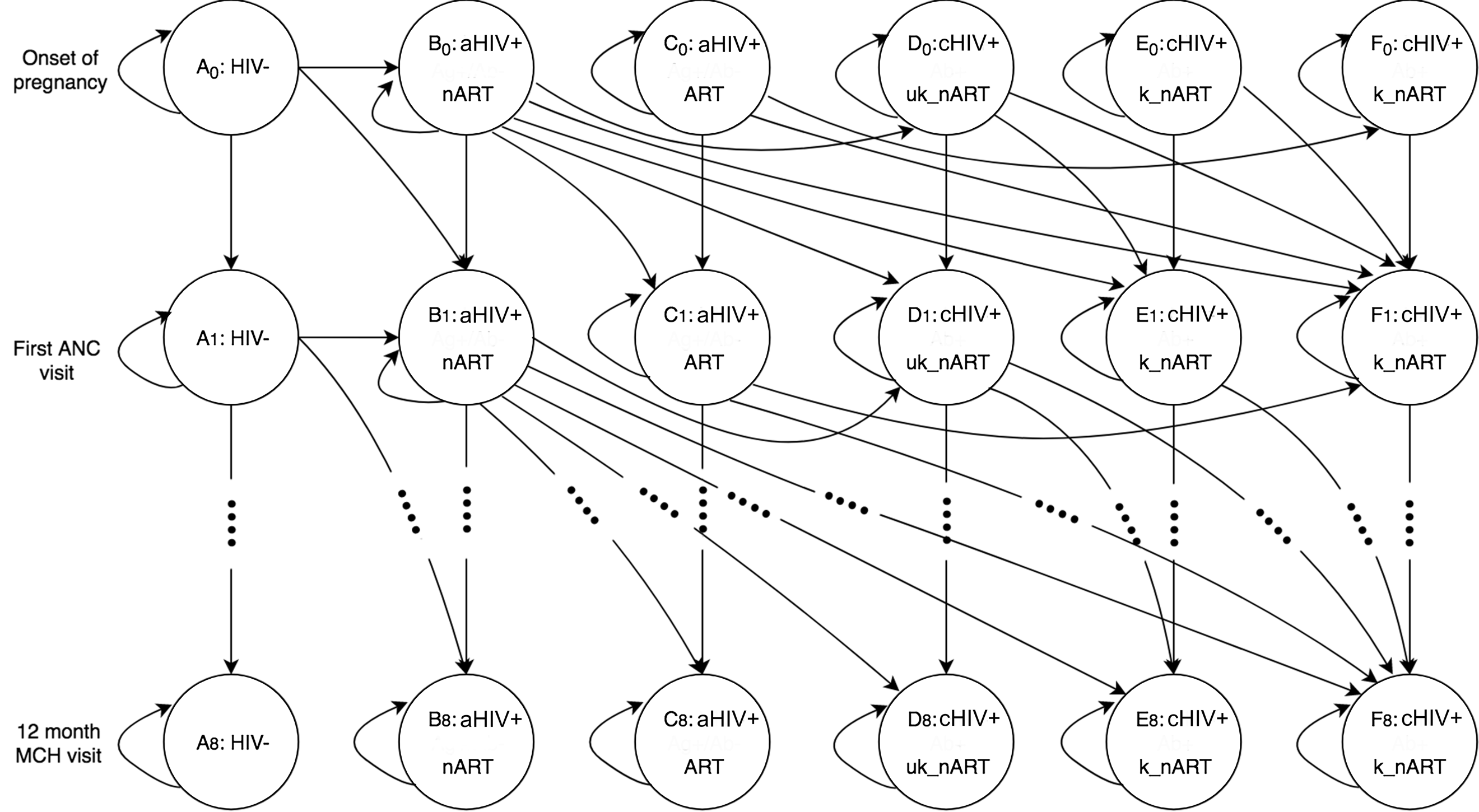


**Figure 1:** Model state-transition diagram. aHIV+ = acute HIV infection; cHIV+ = chronic HIV infection; nART = not on ART; uk = HIV status unknown; k = HIV status unknown

#### 1.1 Transition probability matrices (states)

| To | | | | | | | |
| --- | --- | --- | --- | --- | --- | --- | --- |
|  | | A | B | C | D | E | F |
| From | A: HIV− | $(1-I_{x})$ | $I_{x}$ | 0 | 0 | 0 | 0 |
|  | B: aHIV+, _n_ART | 0 | $\left(1-\frac{1}{d_{a}}\right)$ | 0 | $\left(\frac{1}{d_{a}}\right)$ | 0 | 0 |
|  | C: aHIV+, ART | 0 | $1-ret$ | $\left(1-\frac{1}{d_{a}}\right)*ret$ | 0 | 0 | $\left(\frac{1}{d_{a}}\right)*ret$ |
|  | D: cHIV+, uk_n_ART | 0 | 0 | 0 | 1 | 0 | 0 |
|  | E: cHIV+, k_n_ART | 0 | 0 | 0 | 0 | 1 | 0 |
|  | F: cHIV+, ART | 0 | 0 | 0 | 0 | $1-ret$ | $ret$ |

**Table A2a:** Within stage (between visit) transition probability matrix. All transition probabilities are also multiplied by $(1-\mu_{w})$ in stages 0-2 and 5-7, or by $(1-\mu_{m})$ in stages 3 and 4, where$\mu_{w}$ is the weekly mortality rate of women of reproductive age, and $\mu_{m}$ is the weekly maternal mortality rate at parturition and early post-partum. Stage 0: onset of pregnancy to first ANC; stage 1: first ANC to late ANC; stage 2: late ANC to delivery; stage 3: delivery; stage 4: first 6 weeks postpartum; stage 5: six to 14 weeks postpartum; stage 6: 14 weeks to six months postpartum; stage 7: six to nine months postpartum; stage 8: nine to 12 months postpartum. Abbreviations: $aHIV$= recent HIV infection, $cHIV$= established HIV infection, $uk$= unknown HIV status, $k$= known HIV status,${}_{n}ART$= not receiving antiretroviral treatment (ART),$ART$= receiving ART,$I_{x}$= weekly maternal HIV incidence in model period x, $d_{a}$= duration of recent infection, $ret$= proportion of women retained on ART each week.

| To | | | | | | | |
| --- | --- | --- | --- | --- | --- | --- | --- |
|  |  | A: HIV- | B: aHIV+, _n_ART | C: aHIV+, ART | D: cHIV+, uk_n_ART | E: cHIV+, k_n_ART | F: cHIV+, ART |
| From | A: HIV− | $(1-I_{x})$ | $I_{x}$ | 0 | 0 | 0 | 0 |
|  | B: aHIV+, _n_ART | 0 | $\left(1-\frac{1}{d_{a}}\right)*(1-a_{n}ts_{n}s_{a}rA)$ | $\left(1-\frac{1}{d_{a}}\right)*(a_{n}ts_{n}s_{a}rA)$ | $\left(\frac{1}{d_{a}}\right)*(1-a_{n}ts_{n}s_{c}r)$ | $\left(\frac{1}{d_{a}}\right)*(a_{n}ts_{n}s_{c}r)*(1-A)$ | $\left(\frac{1}{d_{a}}\right)*(a_{n}ts_{n}s_{c}r)*A$ |
|  | C: aHIV+, ART | 0 | 0 | $\left(1-\frac{1}{d_{a}}\right)$ | 0 | 0 | $\left(\frac{1}{d_{a}}\right)$ |
|  | D: cHIV+, uk_n_ART | 0 | 0 | 0 | $(1-a_{n}ts_{n}s_{c}r)$ | $(a_{n}ts_{n}s_{c}r)*(1-A)$ | $(a_{n}ts_{n}s_{c}r)*A$ |
|  | E: cHIV+, k_n_ART | 0 | 0 | 0 | 0 | 1 | 0 |
|  | F: cHIV+, ART | 0 | 0 | 0 | 0 | 0 | 1 |

**Table A2b:** Between stage (at visits) transition probability matrix. All transition probabilities are also multiplied by $(1-\mu_{w})$in stages 0-2 and 5-8, or by $(1-\mu_{m})$in stages 3 and 4. Stage 0: onset of pregnancy to first ANC; stage 1: first ANC to late ANC; stage 2: late ANC to delivery; stage 3: delivery; stage 4: first 6 weeks postpartum; stage 5: six to 14 weeks postpartum; stage 6: 14 weeks to six months postpartum; stage 7: six to nine months postpartum; stage 8: nine to 12 months postpartum. Abbreviations: $aHIV$= recent HIV infection, $cHIV$= established HIV infection, $uk$= unknown HIV status, $k$= known HIV status, ${}_{n}ART$= not receiving antiretroviral treatment (ART), $ART$= receiving ART, $I_{x}$= weekly maternal HIV incidence in model period x, and $d_{a}$= duration of recent infection, $a_{n}$= visit attendance at stage n (ANC visit, MCH visit, or facility delivery), $ts_{n}$ = probability of being tested at visit corresponding to stage n, $s_{a}$= sensitivity of rapid screening test in recent infection, $s_{c}$= sensitivity of rapid screening test in established infection, $A$ = probability of accepting ART if confirmed HIV positive, $r$= probability of receiving results if testing positive.

#### 1.2 Derived parameters

Maternal HIV incidence in model period x is derived by:

(1.1) $I_{x}=incidence_{x}*[1-(red_{prep}*p_{prep})]$

Where $incidence_{x}$is weekly maternal HIV incidence in model period x, $red_{prep}$ is the expected reduction in incidence due to pre-exposure prophylaxis (PrEP) use, and$p_{prep}$ is the probability of fully-adherent PrEP use.

The probability of being tested at the visit corresponding to stage n is derived by:

(1.2) $ts_{n}=(test_{prob}*(1-stockout)*test_{accept})$

Where $test_{prob}$is the probability of the test being offered at the visit corresponding to stage n (set by the model user), $stockout$is the probability of test kit stockout, and $test_{accept}$is the probability the patient consents to testing. At delivery (n=3), $ts_{3}$ is multiplied by $1-ts_{2}$ to reflect that testing occurs at late gestation (late ANC, n=2), not both. Similarly, at six weeks postpartum (n=4), $ts_{4}$ is multiplied by $1-\left( \left( 1-ts_{2} \right)*ts_{3} \right)$ to reflect that testing only occurs at six weeks postpartum only if it did not occur at the previous two time points.

The duration of recent infection is derived by:

(1.3) $d_{a}=d_{Ag-}+d_{Ag+/Ab-}+d_{Ab+/hVL}$

Where $d_{Ag-}$is the duration of the antigen-negative period, $d_{Ag+/Ab-}$is the duration of the antigen-positive, antibody-negative period, and $d_{Ab+/hVL}$ is the duration of the antibody-positive (post-seroconversion) period before set-point viral load is reached.

All other parameters are defined as detailed in Appendix B: Model Parameters.

## 2 Starting states (stage 0)

#### No women are assumed to be in state C (HIV positive, recent infection, on ARTs) at the start of the model period.

#### 2.1 State A (HIV negative)

The number of women who start their pregnancy (and thus the model period) in State A is given as

(2.1) $A_{0}=N[\left( 1-prev_{preg} \right)-\left( I_{early_{preg}}*d_{a} \right)]$

Where$prev\_preg$is the prevalence of HIV among women at the onset of pregnancy, and$N$is the total population size.

#### 2.2 State B (HIV positive, recent infection, not on ARTs)

The number of women who their pregnancy in State B is given as

(2.2) $B_{0}=I_{early\_preg}*d_{a}*N$

Where $I_{early\_preg}$ is the weekly incidence of HIV among women in early pregnancy, and $d_{a}$ is the duration of recent infection.

#### 2.3 State D (HIV positive, established infection, not on ARTs, unknown status)

The number of women who start their pregnancy in State D is given as

(2.3) $D_{0}=prev\_preg*(1-status\_known)*\mathrm{N}$

Where$status\_known$is the probability an HIV positive woman at the start of pregnancy knows her status.

#### 2.4 State E (HIV positive, established infection, not on ARTs, known status)

The number of women who start their pregnancy in State E is given as

(2.4) $E_{0}=prev\_preg*status\_known*(1-A)*\mathrm{N}$

Where *A* is the probability of accepting ART if confirmed HIV positive.

#### 2.5 State F (HIV positive, established infection, on ARTs)

The number of women who start the model period in State F is given as

(2.5) $F_{0}=prev\_preg*status\_known*A*\mathrm{N}$

Where *A* is the probability of accepting ART if confirmed HIV positive.

## 3 Infant transmission

Potential mother-to-child transmission is assumed to occur from all women in an HIV positive state (states B-F). For all equations that follow, S indexes state (A-F), and n indexes stage (0-8). For instance X_F2_ refers to variable X pertaining to state F (established infection, on ARTs), stage 2 (after late ANC, prior to delivery). Maternal deaths at each time step enter an absorbing state, to which none of the following equations are applied. This model assumes no miscarriages occur and that all pregnancies are singleton. To reflect time from testing to ART initiation as well as time from ART initiation to viral suppression, the model assumes no viral suppression among mothers with recent HIV infection on ART.

#### 3.1 *In utero* (stages 0-2)

The number of infant infections generated by women in states B or C$(S\in\{B,C\})$at stage n of pregnancy$(\mathrm{n}\in\{0,1,2\})$is given as

(3.1.1) $Inf_{utero\_Sn}=\left( 1-e^{-\lambda_{utero\_a}*t_{n}} \right)*(N_{Sn}-Inf_{Sn-1});S\in\{B,C\},n\in\{0,1,2\}$

Where $\lambda_{utero\_a}$is the weekly incidence of *in utero* infant transmission from a recently infected mother, $t_{n}$is duration of stage n (in person-weeks),$N_{Sn}$ is the number of women in stage n and state S, and $Inf_{Sn-1}$ is the number of infants infected in the same state S but the previous stage (n-1).

The number of infant infections generated by women in states D or E at stage n of pregnancy$(\mathrm{n}\in\{0,1,2\})$is given as

(3.1.2) $Inf_{utero\_Sn}=\left( 1-e^{-\lambda_{utero\_c}*t_{n}} \right)*(N_{Sn}-Inf_{Sn-1});S\in\{D,E\},n\in\{0,1,2\}$

Where $\lambda_{utero\_c}$is the weekly incidence of *in utero* infant transmission from a mother with an established infection.

#### The number of infant infections generated by women in state F at stage n of pregnancy$(\mathrm{n}\in\{0,1,2\})$is given as

#### (3.1.3) $\boldsymbol{In}\boldsymbol{f}_{\boldsymbol{utero\_Sn}}\boldsymbol{=}\left( \boldsymbol{1-}\boldsymbol{e}^{\boldsymbol{-(}\boldsymbol{\lambda}_{\boldsymbol{utero\_c}}\boldsymbol{[1-(}\boldsymbol{V}_{\boldsymbol{re}\mathbf{d}}\boldsymbol{*}\boldsymbol{p}_{\boldsymbol{VL}}\boldsymbol{)])*}\boldsymbol{t}_{\boldsymbol{n}}} \right)\boldsymbol{*}\left( \boldsymbol{N}_{\boldsymbol{Sn}}\boldsymbol{-In}\boldsymbol{f}_{\boldsymbol{Sn-1}} \right)\boldsymbol{;S\in}\left\{ \boldsymbol{F} \right\}\boldsymbol{, n\in\{0,1,2\}}$

Where $V_{red}$is the proportional reduction in infant transmission incidence attributable to maternal viral load suppression (undetectable viral load), and$p_{VL}$is the probability that a woman receiving ARTs has achieved viral load suppression.

#### 3.2 Delivery and early post-partum (stages 3 and 4)

The number of infant infections generated by women in states B or D at delivery (parturition; n=3) or the first six weeks postpartum (n=4) is given as

(3.2.1) $Inf_{part\_Sn}=\left( 1-e^{-(\lambda_{part\_y}*t_{n})} \right)*(N_{Sn}-Inf_{Sn-1})*(1-\mu_{ne});S\in\{B,D\},n\in\{3,4\}$

Where $\lambda_{part\_y}$ is the weekly incidence of infant transmission at parturition and early post-partum from a mother with recent infection if y=a, and established infection if y=c; $t_{n}$is the duration of model stage n in person-weeks; and$\mu_{ne}$is the probability of neonatal death.

The number of infant infections generated by women in states C or E at delivery (parturition; n=3) is given as

(3.2.2) $Inf_{part\_Sn}=\left( 1-e^{-(\lambda_{part\_y}*[1-(ARV_{red}*p\_ARV)]*t_{n})} \right)*(N_{Sn}-Inf_{Sn-1})*(1-\mu_{ne});S\in\{C,E\},n\in\{3\}$

Where $ARV_{red}$is the proportional reduction in infant transmission incidence attributable to infant antiretroviral (ARV) prophylaxis, and$p\_\mathrm{ARV}$is the probability the infant receives ARV prophylaxis.

The number of infant infections generated by women in states C or E at during the first six weeks postpartum (n=4) is given as

(3.2.3) $\begin{aligned} Inf_{part\_Sn}=\left( 1-e^{-(\lambda_{part\_y}*[1-(ARV_{red}*p\_ARV)]*[1-(nbf_{red}*p\_nbf_{e})]*t_{n})} \right) \\ *(N_{S4}-Inf_{S4-1})*(1-\mu_{ne});S\in\{C,E\},n\in\{4\} \end{aligned}$

Where$nbf_{red}$is the proportional reduction in infant transmission incidence attributable to not breastfeeding and$p\_nbf_{e}$ is the probability of complete breastfeeding avoidance in the early postpartum period.

The number of infant infections generated by women in state F at delivery (parturition; n=3) is given as

(3.2.4) $\begin{aligned} I_{part\_Sn}=\left( 1-e^{-(\lambda_{part\_c}*[1-(ARV_{red}*p\_ARV)]*[1-(V_{red}*p_{VL})]*t_{n})} \right)*(N_{Sn}-I_{Sn-1})*(1-\mu_{ne}); \\ S\in\{F\},n\in\{3\} \end{aligned}$

And the number of infant infections generated by women in state F during the first six weeks postpartum (n=4) is given as

(3.2.5) $\begin{aligned} I_{part\_Sn}=\left( 1-e^{-(\lambda_{part\_c}*[1-(ARV_{red}*p\_ARV)]*[1-(V_{red}*p_{VL})]*[1-(nbf_{red}*p\_nbf_{e})]*t_{n})} \right) \\ *(N_{Sn}-I_{Sn-1})*(1-\mu_{ne});S\in\{F\},n\in\{4\} \end{aligned}$

#### 3.3 Mid and late post-partum (stages 5-8)

The number of infant infections generated by women in states B or D and stage n (n>4) post-partum is given as

(3.3.1) $\begin{aligned} Inf_{pp\_Sn}=\left( 1-e^{-(\lambda_{pp\_x\_y}*[1-(nbf_{red}*p\_nbf_{xk})]*t_{n})} \right)*(N_{Sn}-Inf_{Sn-1})*(1-\mu_{z}); \\ S\in\{B,D\},n\in\{5,6,7,8\} \end{aligned}$

Where $\lambda_{pp\_\mathrm{x}\_y}$ is the weekly incidence of infant transmission in model period x (x=m, mid postpartum; x=l, late postpartum) from a mother who is exclusively breastfeeding and has recent infection if y=a, or established infection if y=c;$p\_nbf_{x}$ is the probability of complete breastfeeding avoidance in model period x, HIV status k (no known infection= states A, B, and D; known infection= states C, E, and F);$\mu_{z}$is the probability of neonatal (z=ne, up to 6 weeks post-partum) or infant (z=i, 6 weeks to 12 months post-partum) death.

The number of infant infections generated by women in states C or E and stage n (n>4) post-partum is given as

(3.3.2) $\begin{aligned} Inf_{pp\_Sn}=\left( 1-e^{-(\lambda_{pp\_x\_y}*[1-(ARV_{red}*p\_ARV)]*[1-(nbf_{red}*p\_nbf_{xk})]*t_{n})} \right)* \\ (N_{Sn}-Inf_{Sn-1})*(1-\mu_{z});S\in\{C,E\},n\in\{5,6,7,8\} \end{aligned}$

The number of infant infections generated by women in state F and stage n (n>4) post-partum is given as

(3.3.3) $\begin{aligned} Inf_{pp\_Sn}=\left( 1-e^{-(\lambda_{pp\_x\_c}*[1-(ARV_{red}*p\_ARV)]*[1-(nbf_{red}*p\_nbf_{xk})]*[1-(V_{red}*p_{VL})]*t_{n})} \right) \\ *(N_{Sn}-Inf_{Sn-1})*(1-\mu_{z});S\in\{F\},n\in\{5,6,7,8\} \end{aligned}$

Note that if the user does not have access to parameters that allow disaggregation of breastfeeding practice by HIV status or doesn’t feel this is appropriate, they may enter the same probability of breastfeeding practice across HIV positive and HIV negative women, effectively collapsing over the k subscript for $p\_nbf_{xk}$.

### 4 Costs

#### 4.1 Maternal testing costs

Testing is assumed to be linked to ANC and MCH visits that would occur in the absence of testing, thus only incremental costs associated with testing at a given visit are modeled. For visits at which testing is offered (defined by the model user), testing occurs in the first time step of that stage ($\tau_{in}$ where *i*=1 and n is defined by testing strategy); that is, if testing will be offered at visit n=3 under a given treatment strategy, testing is assumed to occur at the first time step (week) of stage 3 ($\tau_{13}$).

Testing costs are only applied to women with negative or unknown HIV status, that is women in states A, B, and D. The test set is defined as the total number of women across the three testing states (A, B, or D) at the first time step ($\tau_{1n}$) of a visit (*n*) where testing occurs. It is assumed that women who are tested at late gestation (n=2) are not also tested at delivery (n=3), and that women who are tested at late gestation or delivery are not eligible for retesting at six weeks postpartum (n=4). Thus, the marginal probability of testing at delivery is the product of the conditional probability of testing at delivery and the marginal probability of not testing at late gestation, and the marginal probability of testing at six weeks postpartum is the product of the conditional probability of testing at six weeks postpartum and the marginal probability of not testing at the previous two time points.

#### 4.1a All negative/unknown status states (A, B, and D)

Total costs for screening women at stage n independent of HIV status is given as

(4.1.1) $C_{screen\_Sn}=a_{n}*ts_{n}*screen*N_{test\_\tau_{1n}};S\in\{A,B,D\},n\in\{1,2,...,8\}$

Where $screen$ is the total cost of screening using test per woman tested, and $N_{test\_\tau_{1n}}$is the number of women in the test set at time step $\tau_{1n}$.

Inputs for the total cost of a screening test may include:

- Test kit costs
- Other supplies including gloves, capillary tubes, alcohol swabs, etc.
- Staff and administrative costs above that required for the existing visit (ANC, delivery, or PNC)

#### 4.1b HIV negative women (State A)

Additional testing costs for HIV negative women at stage n are given as

(4.1.2) $C_{false\_pos\_Sn}=a_{n}*ts_{n}*(1-spec)*false\_pos*N_{A\_\tau_{1n}};S\in\{A\},n\in\{1,2,...,8\}$

Where $spec$ is the specificity of the screening test,$N_{A\_\tau_{1n}}$is the number of women in state A at time step$\tau_{1n}$, and$false\_pos$is the cost of a false positive test above that incurred for screening.

Inputs for cost of a false positive test may include:

- Confirmatory and tie-breaker testing
- Additional staff and facility costs

Thus, total testing costs for women in state A at stage n are given as:

(4.1.3) $C_{Sn}=a_{n}*ts_{n}[screen+((1-spec)*false\_pos)]*N_{A\_\tau_{1n}};S\in\{A\},n\in\{1,2,..,8\}$

#### 4.1c HIV positive women (States B and D)

Additional costs for HIV positive women are given as:

(4.1.4) $C_{true\_pos\_Sn}=a_{n}*ts_{n}*sens*r*true\_pos*N_{S\_\tau_{1n}};S\in\{B,D\},n\in\{1,2,..,8\}$

Where$N_{S\_\tau_{1n}}$is the number of women in state S at time step $\tau_{1n}$, and $true\_pos$is the cost of a true positive test above that incurred for screening.

Inputs for the cost of a true positive test may include:

- Confirmatory and tie-breaker testing
- Staff and facility costs associated with additional counseling and linkage to care

Additionally, women newly diagnosed at stages 3 (delivery) or later are offered infant prophylaxis at the time of their diagnosis.

Thus, the total costs for women in states B or D at stage n where n <3 are given as:

(4.1.5) $C_{Sn}=a_{n}*ts_{n}[screen+(sens*r*true\_pos)]*N_{S\_\tau_{1n<3}};S\in\{B,D\},n\in\{0,1,2\}$

And, the total costs for women in states B or D at stage n where n ≥ 3 are given as:

(4.1.6) $\begin{aligned} C_{Sn}=a_{n}*ts_{n}[screen+(sens*r*(true\_pos+(\cos t_{ARV}*p\_ARV))]*N_{S\_\tau_{1n\geq3}}; \\ S\in\{B,D\},n\in\{3,4,...,8\} \end{aligned}$

Where $\cos t_{arv}$is the total cost of a full course of infant prophylaxis (the cost of an entire is course applied at one time, not per week).

#### 4.1d Women in a known status state (C, E, and F)

At delivery (stage n=3) women with known HIV infection (states C, E, and F) are offered ARVs for infant prophylaxis. Women in states C and F also incur costs associated with maternal ART.

Thus, total costs for women in state C or F at stage n≠ 3 are given as:

(4.1.7) $\mathrm{C}_{Sn}=\cos t_{ART}*N_{Sn_{\neq3}}*t_{n\neq3};S\in\{C,F\},n\in\{1,2,4,...,8\}$

Where $\cos t_{art}$ is the total cost of maternal ARTs per week,$N_{Sn_{\neq3}}$is the number of women state S and stage n≠ 3, and $t_{n\neq3}$ is the duration of stage n (where n≠3) in weeks.

Total costs for women in state C or F at stage n=3 are given as:

(4.1.8) $C_{Sn}=[(\cos t_{ARV}*p\_ARV)+(\cos t_{ART}*p\_ART)]*N_{Sn}*t_{n};S\in\{C,F\},n\in\{3\}$

And total costs for women in state E at stage n=3 are given as:

(4.1.9) $C_{Sn}=(\cos t_{ART}*p\_ARV)*N_{Sn}*1;S\in\{E\},n\in\{3\}$

Note, women in state E do not incur costs at any other time stages.

#### 4.2 Maternal pre-exposure prophylaxis (PrEP) costs

Women in states A and B are also eligible to receive PrEP. PrEP costs for women in states A and B at stage n are given as:

(4.2.1) $C_{PrEP\_Sn}=0\leq n\leq8:(\cos t_{\Pr EP}*p\_prep)*N_{Sn}*t_{n};S\in\{A,B\},n\in\{1,2,...,8\}$

Where $\cos t_{\Pr EP}$is the per-week cost of pre-exposure prophylaxis;$p\_prep$is the probability of PrEP reciept and adherence;$N_{Sn}$is the number of women in state S at stage n; and $t_{n}$is the duration of stage n in weeks.

#### 4.3 Pediatric treatment costs

Pediatric treatment costs were calculated over a 20-year time horizon (from birth through age 19) assuming an annual discount rate of 3%.

For an HIV-infected child of age a, $a\in\{1,\ldots,19\}$ the annual cost of ART was calculated as follows:

$$C_{i\_a}=\frac{{cost}_{ART}*p_{ART\_a}*52}{{(1+r)}^{a+1}}$$

Where ${cost}_{ART}$ is the weekly cost of ART, $p_{ART\_a}$is the probability that an infected child of age a receives ART, and r is the discount rate.

For an HIV-infected infant under one year of age infected in stage k, $k\in\{1,2,\ldots8\}$ the annual cost of ART was calculated as follows:

$$C_{i\_0\_k}=\frac{p_{k}{*cost}_{ART}*p_{ART\_a}*52}{{(1+r)}^{a+1}}$$

Where $p_{k}$ is the proportion of the infant’s first year of life remaining after infection in stage k (e.g., 1 for infants infected during pregnancy, 0.5 for infants infected at 6 months).

The total HIV treatment costs over a 20-year time horizon for infants infected through mother-to-child transmission were calculated as:

$$C_{i\_tot}=\sum_{k=1}^{8} C_{i\_0\_k}N_{i\_0\_k}+\sum_{a=1}^{19} C_{a}N_{i\_a}$$

Where $N_{i\_0\_k}$ indicates the number of HIV-infected infants infected in stage k and $N_{i\_a}$ indicates the number of HIV-infected children alive at age a.

#### 4.4 Total costs

Total costs were calculated by summing over maternal testing costs, maternal treatment costs, maternal pre-exposure prophylaxis costs, and pediatric treatment costs.

### 5 Deaths

#### 5.1 Mortality probabilities

The probability of HIV-specific death from age a to a+n conditional on surviving to age a was calculated as follows:

$${}_{n}q_{a\_hiv\_s}=\left( 1-p_{ART}*p_{adh} \right)*{}_{n}q_{a\_hiv\_ntx}$$

Where ${}_{n}q_{a\_hiv\_ntx}$ is the probability of HIV-specific death without ART from age a to a+n conditional on surviving to age a, and $p_{adh}$ is the proportion of HIV-infected children on treatment who are adherent to ART. ${}_{n}q_{a\_hiv\_ntx}$ was derived from cumulative mortality estimates of 32.5% by age 1 and 52.5% by age 2 among HIV-infected infants not receiving ART(1). We assumed no additional HIV-specific mortality after age 2.

The total probability of death from age a to a+n among children infected with HIV was calculated as:

$${}_{n}q_{a\_hiv}=1-\left( 1-{}_{n}q_{a\_hiv\_s} \right)*(1-{}_{n}q_{a_{nhiv}})$$

Where ${}_{n}q_{a_{nhiv}}$is the probability of non-HIV death from age a to a+n conditional on surviving to age a and was derived from country-specific WHO life tables (2). Note that ${}_{n}q_{a_{nhiv}}=0$ for HIV-uninfected children.

Finally, the annual mortality probabilities for HIV-infected and HIV-uninfected children was calculated as follows:

$${}_{1}q_{a\_hiv}=1-exp\{\frac{ln( 1-{}_{n}q_{a\_hiv})}{n}\}$$

$${}_{1}q_{a\_nhiv}=1-exp\{\frac{ln( 1-{}_{n}q_{a\_nhiv})}{n}\}$$

Where *n* indicates the length of the age intervals defined in the WHO life tables (eg, ${}_{4}q_{1}$ corresponds to an interval length of n=4).

**5.2 Deaths**

The population of HIV-infected and HIV-uninfected children was modeled over a 20-year time horizon. At a specific age $a\in\{0,\ldots19\}$, the number of children alive is given as follows:

$$N_{hiv_{a}}=N_{hiv_{a-1}}-N_{hiv_{a-1}}*{}_{1}q_{a-1\_hiv}$$

$$N_{nhiv_{a}}=N_{nhiv_{a-1}}-N_{nhiv_{a-1}}*{}_{1}q_{a-1\_nhiv}$$

The cumulative number of deaths by age $a\in\{0,\ldots19\}$ was calculated as follows:

$$D_{a}=D_{a-1}+N_{hiv_{a-1}}*{}_{1}q_{a-1_{hiv}}+N_{nhiv_{a-1}}*{}_{1}q_{a-1\_nhiv}$$

### 6 Disability-adjusted life years (DALYs)

DALYs incurred by children born to mothers in the model over a 20-year time horizon were discounted annually at 3%.

**6.1 Years of life lost (YLLs)**

Discounted YLLs incurred at age $a\in\{0,\ldots19\}$ were calculated as follows:

$${YLL}_{a}=\frac{D_{a}}{{(1+r)}^{a+1}}$$

**6.2 Years lived with disability (YLDs)**

Discounted YLDs incurred at age $a\in\{0,\ldots19\}$ were calculated as follows:

$${YLD}_{a}=\frac{N_{hiv_{a}}*[p_{ART}*p_{adh}*{dw}_{HIV_{tx}}+\left( 1-p_{ART}*p_{adh} \right)*({dw}_{HIV_{ntx}}*{pLE}_{HIV}+{dw}_{{AIDS}_{ntx}}*\left( 1-{pLE}_{HIV} \right))}{{(1+r)}^{a+1}}$$

Where ${dw}_{HIV_{tx}}$ is the disability weight for HIV-infected individuals on ART, ${dw}_{HIV_{ntx}}$ is the disability weight for HIV-infected individuals not on ART without AIDS, ${dw}_{{AIDS}_{ntx}}$ is the disability weight for HIV-infected individuals not on ART with AIDS, and ${pLE}_{HIV}$ is the proportion of the natural history of HIV prior to AIDS.

**6.3 DALYs**

Total DALYs were calcualted by summing YLDs and YLLs over all ages.

$$DALYs=\sum_{a=0}^{19} {YLD}_{a}+{YLL}_{a}$$

## 7 References

1. Newell ML, Coovadia H, Cortina-Borja M, Rollins N, Gaillard P, Dabis F, et al. Mortality of infected and uninfected infants born to HIV-infected mothers in Africa: a pooled analysis. Lancet. 2004;364(9441):1236-43.

2. World Health Organization. Life tables by country. Global Health Observatory data repository. Geneva [Available from: <http://apps.who.int/gho/data/view.main.61540?lang=en>.
