# Supplementary material for: Optimizing HIV retesting during pregnancy and postpartum in four countries: a cost‐effectiveness analysis
Source: J Int AIDS Soc. 2021 Mar 31;24(4):e25686. doi: 10.1002/jia2.25686 (PMC8010369; doi:10.1002/jia2.25686)
Supplement: Supplementary file 4 — Appendix S3. Model parameters [file JIA2-24-e25686-s007.docx]

**Appendix 3: Model Parameters**

**1 Maternal state transitions**

**1.1 Static across countries**

| *Parameter* | *Value* | | *Data year(s); Source* |  |  |
| --- | --- | --- | --- | --- | --- |
| Sensitivity of rapid screening test, Ag- | 0 | | Assumed |  |  |
| Sensitivity of rapid screening test, Ag+/Ab- | 0 | | Assumed |  |  |
| Sensitivity of rapid screening test, Ab+ | 1 | | 2018; WHO PQ Public Report (2018)^1^ |  |  |
| Duration of Ag- period (weeks) | 2.3 | | 2003; Cohen (2010)^2^ |  |  |
| Duration of Ag+/Ab- period (weeks) | 0.7 | | 2003; Cohen (2010)^2^ |  |  |
| Duration of early Ab+ period (higher viral load; weeks) | 6 | | Assumed |  |  |
| Duration of acute maternal HIV infection (weeks) | 9 | | Calculated^a^ |  |  |
| Maternal PrEP use | 0 | | Assumed |  |  |
| Proportion retained in ART at 1 year postpartum^b^ | | 0.732 | 2011-2013; Haas (2016)^3^ | |  |
| Weekly risk of ART dropout | 0.0033 | | Calculated^c^ |  |  |
| Proportional reduction in HIV incidence rate due to fully-adherent PrEP use | 0.71 | | 2008-2011; Heffron (2017)^4^ |  |  |
| Gestational age in weeks, delivery | 39 | | Assumed |  |  |
| Gestational age in weeks, early-postpartum | 6 | | Assumed |  | |
| Gestational age in weeks, mid-postpartum | 14 | | Assumed |  |  |
| Gestational age in weeks, late-postpartum | 26 | | Assumed |  |  |
|  |  | |  |  |  |

**Table A3a:** ^a^Sum of the Ag-, Ag+/Ab-, and early Ab+ durations. ^b^Midpoint of 12 and 24 month retention. ^c^Calculated from proportion retained in ART at 1 year postpartum assuming a constant proportion drops out each week. Abbreviations: Ag-: antigen negative; Ag+: antigen positive; Ab-: antibody negative; Ab+: antibody positive; PrEP: maternal pre-exposure prophylaxis; ANC: antenatal care.

**1.1 Country-specific**

| *Parameter^a^* | *Value* | | | | *Data year(s), Source* | | | |
| --- | --- | --- | --- | --- | --- | --- | --- | --- |
|  | *Kenya* | *South Africa* | *Colombia* | *Ukraine* | *Kenya* | *South Africa* | *Colombia* | *Ukraine* |
| Weekly incidence rate prior to first ANC visit | 0.000331^b^ | 0.000227^b,i^ | 0.00001 | 0.000002^p^ | 2011-2013; Kinuthia (2015)^5^ | 2004-2013; Thomson (2018)^6^, Kinuthia (2015)^5^ | In-country source | 2007-2019; UNAIDS Data (2019)^7,o^ |
| Weekly incidence rate after first ANC and up to delivery | 0.000331^b^ | 0.000739^b,i^ | 0.00002 | 0.000004^p^ | 2011-2013; Kinuthia (2015)^5^ | 2004-2013; Thomson (2018)^6^, Kinuthia (2015)^5^ | Assumed | 2007-2019; UNAIDS Data (2019)^7,o^ |
| Weekly incidence rate first 6 weeks postpartum | 0 | 0 | 0 | 0 | Assumed | Assumed | Assumed | Assumed |
| Weekly incidence rate 6 weeks-12 months postpartum | 0.00269^b^ | 0.0009^b^ | 0.000023 | 0.000003^q^ | 2011-2013; Kinuthia (2015)^5^ | 2004-2013; Thomson (2018)^6^ | Assumed | 2007-2019; UNAIDS Data (2019)^7,o^ |
| Probability of attending first ANC visit | 0.96^c^ | 0.94^c^ | 0.97 | 0.998 | 2012-2014; Kenya 2014 DHS^8^, Sirengo (2014)^9^ | 2016; South Africa 2016 DHS^10^ | In-country source | 2018; Bozicevic (2018)^11^ |
| Probability of attending late ANC visit | 0.93^d^ | 0.78^k^ | 0.88 | 0.90 | 2014; Kenya 2014 DHS^8^ | 2016; South Africa 2016 DHS^10^ | In-country source | Assumed |
| Probability of facility delivery | 0.62 | 0.96 | 0.99 | 0.99 | 2014; Kenya 2014 DHS^8^ | 2016; South Africa 2016 DHS^10^ | In-country source | 2007; Ukraine DHS (2007)^12^ |
| Probability of attending 6 week MCH visit | 0.96^e^ | 0.90^e^ | 0.92^n^ | 0.99 | 2014; Kenya 2014 DHS^8^ | In-country source | 2018; WHO-UNICEF (2018)^13^ | Assumed |
| Probability of attending 14 week MCH visit | 0.88^e^ | 0.73^e^ | 0.87^n^ | 0.93 | 2014; Kenya 2014 DHS^8^ | In-country source | 2018; WHO-UNICEF (2018)^13^ | Assumed |
| Probability of attending 6 month MCH visit | 0.87^f^ | 0.86^e^ | 0.90^n^ | 0.96 | 2014; Kenya 2014 DHS^8^ | In-country source | 2018; WHO-UNICEF (2018)^13^ | Assumed |
| Probability of attending 9 month MCH visit | 0.85^e^ | 0.62^e^ | 0.89^n^ | 0.95 | 2014; Kenya 2014 DHS^8^ | In-country source | 2018; WHO-UNICEF (2018)^13^ | Assumed |
| Probability of HIV test kit stock-out | 0.05 | 0.05 | 0 | 0 | Assumed | Assumed | Assumed | Assumed |
| Probability of HIV test acceptance | 0.84 | 0.98 | 0.89 | 0.97 | 2011; Kohler (2014)^14^ | 2010-2013; Myer (2015)^15^ | In-country source | 2018; Bozicevic (2018)^11^ |
| Probability of receiving results of HIV test | 0.98 | 0.98 | 1 | 1 | 2012-2013; Kohler (2014)^14^ | 2016; South Africa 2016 DHS^10^ | Assumed | Assumed |
| Maternal ART use | 0.91 | 0.87 | 0.88 | 0.95 | 2010-2019; UNAIDS Data (2019)^17^ | 2016-2019; UNAIDS Data (2019)^18^ | 2014-2015; PMTCT report^19^ | 2007-2019; UNAIDS Data (2019)^7^ |
| Virally suppressed^g^ | 0.88 | 0.72^l^ | 0.88 | 0.88 | 1998-2013; Snippenburg (2017)^20^ | 2013-2014; Brittain (2019)^21^ | 1998-2013; Snippenburg (2017)^20^ | 1998-2013; Snippenburg (2017)^20^ |
| Weekly mortality rate for women of reproductive age, during pregnancy^h^ | 0.0001 | 0.0001 | 0.00002 | 0.00002 | 2016; WHO life tables (2016)^22^ | 2016; WHO life tables (2016)^22^ | 2016; WHO life tables (2016)^22^ | 2016; WHO life tables (2016)^22^ |
| Weekly mortality rate, delivery through 6 weeks postpartum^i^ | 0.0006 | 0.0002 | 0.0001 | 0.00003 | 2017; World Bank (2017)^23^ | 2017; World Bank (2017)^23^ | 2017; World Bank (2017)^23^ | 2017; World Bank (2017)^23^ |
| Weekly mortality rate, 6 weeks to 12 months postpartum^h^ | 0.0001 | 0.0001 | 0.00002 | 0.00002 | 2016; WHO life tables (2016)^22^ | 2016; WHO life tables (2016)^22^ | 2016; WHO life tables (2016)^22^ | 2016; WHO life tables (2016)^22^ |
| Gestational age in weeks, first ANC | 22 | 18^m^ | 15 | 10 | 2013; McGrath (2018)^24^ | 2016; South Africa 2016 DHS^10^ | In-country source | Assumed |
| Gestational age in weeks, late ANC | 33 | 36 | 24 | 28 | Assumed | Assumed | Assumed | Assumed |

**Table A3b:** ^a^Incidence rates refer to incidence of HIV in the mother. ^b^Calculated weekly incidence rate during pregnancy from annual rate. ^c^4% reported no ANC visits. ^d^Calculated as 100 minus the percentage who had none/only one visit ^e^Based on vaccine coverage, adjusted down by 2 percentage points to account for assumed vaccination delay. ^f^Calculated as mid-point between 14 wk and 9 mo. ^g^Among women on ART. ^h^Average mortality rate of each age group between 15-49, divided by 52 to get weekly rate. ^i^Calculated weekly rate from maternal mortality ratio. ^j^Calculated as weighted average of 60% from Kinuthia (2015) and 40% from Thomson (2018). ^k^Per South Africa 2016 DHS, 76% had 4 or more ANC visits and 2% had their first ANC in the 3^rd^ trimester. ^l^Percentage of women at 12 months postpartum who had VL ≥ 1000 copies/mL. ^m^Calculated from median months pregnant at first ANC visit ^n^Calculated from vaccination coverage. ^o^Calculated from country-level HIV incidence. ^p^Assumes 1/3 of incident infections during pregnancy occur before first ANC visit and 2/3 between first ANC visit and delivery. ^q^Assumes half of incident infections during postpartum period occur between delivery and 6 months and half between 6 and 12 months.

[1]

**2 Infant transmission**

**2.1 Static across countries**

| *Parameter^a^* | *Value* | *Data year(s); Source* |
| --- | --- | --- |
| Incidence of infant transmission *in utero*, established infection | 0.0019^b^ | 2002; Duri (2010)^25^ |
| Incidence of infant transmission *in utero*, recent infection | 0.0165^c^ | 1997-2000; Marinda (2011)^26^ |
| Incidence of infant transmission, parturition and first six weeks postpartum, established infection, EBF | 0.0226^d^ | 1997-2000; Zijenah (2004)^27^ |
| Incidence of infant transmission, parturition and first six weeks postpartum, recent infection, EBF | 0.0294^e^ | Assumed |
| Incidence of infant transmission, mid post-partum, established infection, EBF | 0.002^f^ | 2001-2003; Coovadia (2007)^28^ |
| Incidence of infant transmission, mid post-partum, recent infection, EBF | 0.0054^g^ | 1994-1999; Liang (2009)^29^ |
| Incidence of infant transmission, late post-partum, established infection, EBF | 0.0005 | Assumed |
| Incidence of infant transmission, late post-partum, recent infection, EBF | 0.0054^g^ | 1994-1999; Liang (2009)^29^ |
| Proportional reduction in transmission due to infant ARV use | 0.675 | 2004-2007; Kumwenda (2008)^30^ |
| Proportional reduction in transmission due to maternal viral load suppression | 0.95 ^h^ | 2004-2006; Kilewo (2009)^31^ |
| Proportional reduction in incidence rate of infant transmission due to NBF | 1 | Assumed |

**Table A3c:** ^a^Incidence: weekly incidence rate. ^b^Cumulative incidence of 7.46%, distributed over 40 weeks of pregnancy using decay formula and converted to rate; viral load suppression: undetectable viral load; NBF: complete breastfeeding avoidance; MF: mixed feeding. ^c^Cumulative incidence of 14.15% distributed over 9 weeks of acute infection and converted to rate. ^d^Cumulative incidence of 16% distributed over 7 weeks corresponding to delivery and first 6 weeks post-partum and converted to rate. ^e^Assumed 30% higher than the established infection. ^f^Six-month cumulative incidence among infants who were negative at 6 weeks (4%) distributed over 20 weeks (6 months-6 weeks) and converted to rate. ^g^Cumulative incidence of 35.8% among women infected at delivery distributed over 66 weeks duration and converted to rate. ^h^Estimate of infant transmission due to viral load supression is 4% at 6 months and 6% at 18 months so midpoint was used for transmission at 1 year. Abbreviations: EBF: Exclusive breastfeeding; early postpartum: first 6 weeks; mid postpartum: 6 weeks-6 months; late postpartum: 6-12 months; ART: antiretroviral treatment; ARV: infant antiretroviral prophylaxis.

**2.2 Country-specific**

| *Parameter* | *Value* | | | | *Data year, Source* | | | |
| --- | --- | --- | --- | --- | --- | --- | --- | --- |
|  | *Kenya* | *South Africa* | *Colombia* | *Ukraine* | *Kenya* | *South Africa* | *Colombia* | *Ukraine* |
| Probability infant receives ARVs | 0.936^a^ | 0.99 | 0.96 | 0.98 | 2013; McGrath (2018)^24^ | In-country source | In-country source | Assumed |
| Probability HIV-infected infants receives ART^b^ | 0.61 | 0.63 | 0.579 | 0.95 | 2010-2019; UNAIDS (2018)^17^ | 2016-2019; UNAIDS (2019)^18^ | 2014-2015; PMTCT Report^19^ | 2007-2019; UNAIDS (2019) ^7^ |
| Probability of NBF early postpartum, HIV- | 0.001 | 0.07^h^ | 0.031 | 0.046 | In-country source | 2015; West (2019)^32^ | 2010-2017; UNICEF^33^ | 2010-2017; UNICEF^33^ |
| Probability of NBF early postpartum, HIV+ | 0.025 | 0.34^h^ | 0.98 | 0.95 | In-country source | 2015; West (2019)^32^ | 2014-2015; PMTCT Report^19^ | Assumed |
| Probability of NBF mid postpartum, HIV- | 0.0058^c^ | 0.19^h^ | 0.016 | 0.023 | Assumed | 2015; West (2019)^32^ | Assumed | Assumed |
| Probability of NBF mid postpartum, HIV+ | 0.21^c^ | 0.45^h^ | 0.98 | 0.99 | Assumed | 2015; West (2019)^32^ | 2014-2015; PMTCT Report^19^ | Assumed |
| Probability of NBF late postpartum, HIV- | 0.009^d^ | 0.42^h^ | 0.0078 | 0.0115 | In-country source | 2015; West (2019)^32^ | Assumed | Assumed |
| Probability of NBF late postpartum, HIV+ | 0.33^d^ | 0.63^h^ | 0.98 | 0.99 | In-country source | 2015; West (2019)^32^ | 2014-2015; PMTCT Report^19^ | Assumed |
| Neonatal mortality, birth-6 weeks | 0.0049^e^ | 0.0029^e^ | 0.0018^e^ | 0.001^e^ | 2017; IHME (2017)^34^ | 2017; IHME (2017)^34^ | 2017; IHME (2017)^34^ | 2017; IHME (2017)^34^ |
| Infant mortality, >6 weeks-12 months | 0.00031^f^ | 0.00025^f^ | 0.00009^f^ | 0.00006^f^ | 2017; IHME (2017)^34^ | 2017; IHME (2017)^34^ | 2017; IHME (2017)^34^ | 2017; IHME (2017)^34^ |
| _1_q_0_, HIV- or HIV+ on ART^i^ | 0.0365 | 0.0335 | 0.013 | 0.0085 | 2016; WHO life tables (2016)^22^ | 2016; WHO life tables (2016)^22^ | 2016; WHO life tables (2016)^22^ | 2016; WHO life tables (2016)^22^ |
| _4_q_1_, HIV- or HIV+ on ART^i^ | 0.0145 | 0.0095 | 0.002 | 0.001 | 2016; WHO life tables (2016)^22^ | 2016; WHO life tables (2016)^22^ | 2016; WHO life tables (2016)^22^ | 2016; WHO life tables (2016)^22^ |
| _5_q_5_, HIV- or HIV+ on ART^i^ | 0.007 | 0.004 | 0.0015 | 0.001 | 2016; WHO life tables (2016)^22^ | 2016; WHO life tables (2016)^22^ | 2016; WHO life tables (2016)^22^ | 2016; WHO life tables (2016)^22^ |
| _5_q_10_, HIV- or HIV+ on ART^i^ | 0.0055 | 0.0065 | 0.0025 | 0.001 | 2016; WHO life tables (2016)^22^ | 2016; WHO life tables (2016)^22^ | 2016; WHO life tables (2016)^22^ | 2016; WHO life tables (2016)^22^ |
| _5_q_15_, HIV- or HIV+ on ART^i^ | 0.0095 | 0.0075 | 0.008 | 0.003 | 2016; WHO life tables (2016)^22^ | 2016; WHO life tables (2016)^22^ | 2016; WHO life tables (2016)^22^ | 2016; WHO life tables (2016)^22^ |
| Survival to 1 year, HIV- | 0.96^g^ | 0.97^g^ | 0.99^g^ | 0.99^g^ | 2016; WHO life tables (2016)^22^ | 2016; WHO life tables (2016)^22^ | 2016; WHO life tables (2016)^22^ | 2016; WHO life tables (2016) ^22^ |
| Survival to 1 year, HIV+ on ART | 0.96^g^ | 0.97^g^ | 0.99^g^ | 0.99^g^ | 2016; WHO life tables (2016)^22^ | 2016; WHO life tables (2016)^22^ | 2016; WHO life tables (2016) ^22^ | 2016; WHO life tables (2016) ^22^ |
| Survival to 1 year, HIV+ not on ART | 0.65 | 0.65 | 0.65 | 0.65 | 1992-2000; Newell (2004)^35^ | 1992-2000; Newell (2004)^35^ | 1992-2000; Newell (2004)^35^ | 1992-2000; Newell (2004)^35^ |
| Survival to 2 years, HIV+ not on ART | 0.475 | 0.475 | 0.475 | 0.475 | 1992-2000; Newell (2004)^35^ | 1992-2000; Newell (2004)^35^ | 1992-2000; Newell (2004)^35^ | 1992-2000; Newell (2004)^35^ |
| Pediatric ART adherence | 0.84 | 0.84 | 0.63 | 0.62 | 1999-2013; Kim (2014)^36^ | 1999-2013; Kim (2014)^36^ | 1999-2013; Kim (2014)^36^ | 1999-2013; Kim (2014)^36^ |
| Adult ART coverage | 0.69 | 0.62 | 0.74 | 0.52 | 2019; UNAIDS (2019)^37^ | 2019; UNAIDS (2019)^37^ | 2019; UNAIDS (2019)^37^ | 2019; UNAIDS (2019)^37^ |

**Table A3d:**

**^a^**Calculated from probability of receiving ARVs conditional on HIV status, weighted by probabilities of HIV status (Table 3: 0.57*(13/188)+0.963*(175/188)). ^b^Based on the percent of infants with early infant diagnosis. ^c^Estimate derived by assuming a linear trend between measured breastfeeding practices at 6 weeks and 9 months. ^d^Values measured at 9 months postpartum were assumed to be representative of the 6-12 month postpartum period. ^e^Weighted average of early neonatal (0-6 days) and late neonatal (7-28 days) mortality rates, converted to daily rate, then converted to weekly probability. ^f^Post-neonatal mortality rate converted to daily rate then weekly probability. ^g^Assumed to be same as general population estimate. ^h^Calculated as percentage not exclusively breastfeeding; assumes no mixed feeding. Abbreviations: NBF: no breastfeeding; early postpartum: first 6 weeks; mid postpartum: 6 weeks-6 months; late postpartum: 6-12 months; ART: antiretroviral treatment;

**3 Maternal starting states**

| *Parameter* | *Value* | | | | *Data year, Source* | | | |
| --- | --- | --- | --- | --- | --- | --- | --- | --- |
|  | *Kenya* | *South Africa* | *Colombia* | *Ukraine* | *Kenya* | *South Africa* | *Colombia* | *Ukraine* |
| Prevalence of HIV | 0.061^a^ | 0.31 | 0.004 | 0.007 | 2010-2017; UNAIDS (2018) ^17^ | 2017; Woldesenbet (2019) ^38^ | In-country source | 2018; Bozicevic (2018)^11^ |
| Probability of HIV-infected pregnant women known HIV status | 0.57^b^ | 0.608 | 0.67 | 0.98 | 2013; Ronen (2017)^39^ | 2017; Woldesenbet (2019) ^38^ | 2011-2012; Pranchniak-rincon (2016)^40^ | Assumed |

**Table A3e:** ^a^Derived from percentage among women ages 15-49. ^b^Calculated by weighted average of adolescents and adults.

**4 Costs**

| *Parameter* | *Value (2017 USD)* | |  | |  | | *Data year, Source* | |  |  |
| --- | --- | --- | --- | --- | --- | --- | --- | --- | --- | --- |
|  | *Kenya* | *South Africa* | *Colombia* | *Ukraine* | | *Kenya* | | *South Africa* | *Colombia* | *Ukraine* |
| Screening, 3rd generation rapid test ^ab^ | 2.64 | 7.72 | 6.68 | 3.99 | | Microcosting* | | Microcosting* and in-country source | Microcosting* and in-country source | Microcosting* and in-country source |
| Additional costs, true-positive screening tests ^b^ | 3.68 | 11.39 | 8.53 | 4.18 | | Microcosting* | | Microcosting* and in-country source | Microcosting* and in-country source | Microcosting* and in-country source |
| Additional costs, false-positive screening tests^b^ | 26.39 | 34.17 | 74.83 | 19.80 | | 2008-2011; Cintron (2017)^41^ and microcosting | | Microcosting* and in-country source | Microcosting* and in-country source | Microcosting* and in-country source |
| Per-week costs of maternal ART | 4.86 | 4.79 | 18.89^e^ | 32.84 | | 2016; Larson (2018)^42^ | | 2000-2016; Meyer-Rath (2019)^43^ | In-country source | 2012; World Bank (2013)^44^ |
| Total cost of a full course of infant ARVs | 2.32^c^ | 3.82 | 52.10^f^ | 4.00 | | 2011-2012; Bautista-Arrendondo (2013)^45^ | | 2011-2012; Bautista-Arrendondo (2013)^45^ | In-country source | In-country source |
| Per week costs of PrEP delivery | 6.19^d^ | 6.19^d^ | 18.89^g^ | 19.38^g^ | | 2017-2018; Roberts (2019)^46^ | | 2017-2018; Roberts (2019)^46^ | In-country source | Model; Alistar (2014)^47^ |
| Per week costs of infant ART, per infant | 6.73 | 5.46 | 18.89^g^ | 32.84^g^ | | 2011; CDC (2013)^48^ | | 2000-2016; Meyer-Rath (2019) | Assumed | Assumed |

**Table A3Sf:** ^a^Inputs include screening test kit, other supply costs, and personnel costs. ^b^Cost applied at every visit. ^c^Drug costs only. ^d^Costs as implemented. ^e^Cost for 9 months of pregnancy in pesos, converted to USD, then converted to weekly cost. ^f^Cost for 42 days in pesos, divided by 6 to get weekly rate, then converted to USD. ^g^Assumed to be the same as maternal ART costs. *Microcosting details provided in Appendix 5. Abbreviations: ART: antiretroviral treatment; ARV: antiretroviral prophylaxis; PrEP: pre-exposure prophylaxis. Testing assumptions: *Kenya*: third generation screening test = Alere Determine™ HIV-1/2; fourth generation screening test= Alere Determine™ HIV-1/2 Ag/Ab Combo; confirmatory test= First Response; tie-breaker test= viral load testing, defined in more detail in reference. *South Africa:* Confirmatory and tie-breaker testing consists of repeat of screening algorithm^49^. Medication assumptions: *Kenya*: maternal ART= twice-daily tenofovir (TDF) + lamivudine (3TC) + efavirenz (EFV); infant ARV = 6 months nevirapine (NVP) and cotrimoxazole (CTX); infant ART= 3TC + zidovudine (AZT) + NVP for infants <2 weeks old, abacavir (ABC) + 3TC + lopinavir/ritonavir (LPV/r) for infants >2 weeks old; *South Africa:* maternal ART= tenofovir (TDF) + 3TC + dolutegravir (DTG); infant ARV= 6 weeks NVP + CTX; infant ART= ABC + 3TC + LPV/r; *Colombia:* maternal ART = ZDV + 3TC + LPV/r; infant ARV = 6 weeks ZDV + 1 dose NVP; *Ukraine:* maternal ART = TDF/emtricitabine (FTC) + EFV; infant ARV = 4 weeks AZT + 4 weeks 3TC + 2 weeks NVP. Inflation adjustment: Bureau of Labor Statistics CPI Inflation Calculator^50^, applied only if reference provides cost estimates for years prior to 2017.

**References**

1. WHO Prequalification of In Vitro Diagnostics Public Report. Product: Alere™ HIV Combo WHO reference number: PQDx 0243-013-00. September 2018, version 4.0. <https://www.who.int/diagnostics_laboratory/evaluations/pq-list/hiv-rdts/180913_amended_final_pqpr_0033_013_00_v6.pdf?ua=1>. Accessed.

2. Cohen MS, Gay CL, Busch MP, Hecht FM. The detection of acute HIV infection. *J Infect Dis.* 2010;202 Suppl 2:S270-277.

3. Haas AD, Tenthani L, Msukwa MT, et al. Retention in care during the first 3 years of antiretroviral therapy for women in Malawi's option B+ programme: an observational cohort study. *The lancet HIV.* 2016;3:e175-182.

4. Heffron R, McClelland RS, Balkus JE, et al. Efficacy of oral pre-exposure prophylaxis(PrEP) for HIV among women with abnormal vaginal microbiota: a post-hoc analysis of the randomized, placebo-controlled Partners PrEP Study. *Lancet HIV.* 2017;4:449-456.

5. Kinuthia J, Drake AL, Matemo D, et al. HIV acquisition during pregnancy and postpartum is associated with genital infections and partnership characteristics. *Aids.* 2015;29(15):2025-2033.

6. Thomson KA, Hughes J, Baeten JM, et al. Increased Risk of HIV Acquisition Among Women Throughout Pregnancy and During the Postpartum Period: A Prospective Per-Coital-Act Analysis Among Women With HIV-Infected Partners. *J Infect Dis.* 2018;218(1):16-25.

7. UNAIDS Country Factsheet. Ukraine. <https://www.unaids.org/en/regionscountries/countries/ukraine>. Published 2019. Accessed January 14, 2020.

8. *Kenya National Bureau of Statistics, Kenya Ministry of Health, Kenya National AIDS Control Council,Kenya Medical Research Institute, and Kenya National Council for Population and Development. Kenya Demographic and Health Survey 2014.* Rockville, MD, USA 2015.

9. Sirengo M, Muthoni L, Kellogg TA, et al. Mother-to-child transmission of HIV in Kenya: results from a nationally representative study. *Journal of acquired immune deficiency syndromes (1999).* 2014;66 Suppl 1:S66-74.

10. *National Department of Health (NDoH), Statistics South Africa (Stats SA), South African Medical Research Council (SAMRC), and ICF. South Africa Demographic and Health Survey 2016: Key Indicators.* Pretoria, South Africa, and Rockville, Maryland, USA 2017.

11. Bozicevic ID, Z. Report on Pre-Validation of Elimination of Mother-to-Child Transmission of HIV in Ukraine. December 2018.

12. DHS Program. Ukraine Demographic and Health Survey. <https://dhsprogram.com/publications/publication-FR210-DHS-Final-Reports.cfm>. Published 2007. Accessed January 16, 2020.

13. World Health Organization. WHO-UNICEF Estimates of DTP1 Coverage. <http://apps.who.int/immunization_monitoring/globalsummary/timeseries/tswucoveragedtp1.html>. Published 2018. Accessed January 26, 2021.

14. Kohler PK, Okanda J, Kinuthia J, et al. Community-based evaluation of PMTCT uptake in Nyanza Province, Kenya. *PLoS One.* 2014;9(10):e110110.

15. Myer L, Phillips T, Manuelli V, McIntyre J, Bekker LG, Abrams EJ. Evolution of antiretroviral therapy services for HIV-infected pregnant women in Cape Town, South Africa. *Journal of Acquired Immune Deficiency Syndrome.* 2015.

16. UNAIDS Country Factsheets. Kenya. <https://www.unaids.org/en/regionscountries/countries/kenya>. Published 2019. Accessed January 27, 2020.

17. UNAIDS Country Factsheets. South Africa. <https://www.unaids.org/en/regionscountries/countries/southafrica>. Published 2019. Accessed January 27, 2020.

18. *ETMI - PLUS: Estrategia Nacional para la Eliminación de la Transmisión Materno Infantil del VIH, la sífilis congénita, la hepatitis B y la enfermedad de Chagas. Comportamiento de la Transmisión Materno Infantil del VIH en Colombia. Medición de la Cohorte 2017. Dirección de Promoción y Prevención Grupo de Sexualidad, Derechos Sexuales y Derechos Reproductivos. Colombia 29 de mayo de 2019*

20. Snippenburg W, Nellen F, Smit C, Wensing A, Godfried MH, Mudrikova T. Factors associated with time to achieve an undetectable HIV RNA viral load after start of antiretroviral treatment in HIV-1-infected pregnant women. *J Virus Erad.* 2017;3(1):34-39.

21. Brittain K, Mellins CA, Remien RH, et al. Impact of HIV-status disclosure on HIV viral load in pregnant and postpartum women on antiretroviral therapy. *JAIDS Journal of Acquired Immune Deficiency Syndromes.* 2019:1.

22. World Health Organization. Country Life Tables. <http://apps.who.int/gho/data/?theme=main&vid=60850>. Published 2016. Accessed2019.

23. World Bank. Maternal mortality ratio modeled estimates, 2017. <https://data.worldbank.org/indicator/sh.sta.mmrt>. Published 2019. AccessedFebruary 28, 2019.

24. McGrath CJ, Singa B, Langat A, et al. Non-disclosure to male partners and incomplete PMTCT regimens associated with higher risk of mother-to-child HIV transmission: a national survey in Kenya. *AIDS Care.* 2018;30(6):765-773.

25. Duri K, Gumbo FZ, Kristiansen KI, et al. Antenatal HIV-1 RNA load and timing of mother to child transmission; a nested case-control study in a resource poor setting. *Virol J.* 2010;7:176.

26. Marinda ET, Moulton LH, Humphrey JH, et al. In utero and intra-partum HIV-1 transmission and acute HIV-1 infection during pregnancy: using the BED capture enzyme-immunoassay as a surrogate marker for acute infection. *Int J Epidemiol.* 2011;40(4):945-954.

27. Zijenah LS, Moulton LH, Iliff P, et al. Timing of mother-to-child transmission of HIV-1 and infant mortality in the first 6 months of life in Harare, Zimbabwe. *Aids.* 2004;18(2):273-280.

28. Coovadia HM, Rollins NC, Bland RM, et al. Mother-to-child transmission of HIV-1 infection during exclusive breastfeeding in the first 6 months of life: an intervention cohort study. *Lancet.* 2007;369(9567):1107-1116.

29. Liang K, Gui X, Zhang YZ, Zhuang K, Meyers K, Ho DD. A case series of 104 women infected with HIV-1 via blood transfusion postnatally: high rate of HIV-1 transmission to infants through breast-feeding. *J Infect Dis.* 2009;200(5):682-686.

30. Kumwenda NI, Hoover DR, Mofenson LM, et al. Extended antiretroviral prophylaxis to reduce breast-milk HIV-1 transmission. *N Engl J Med.* 2008;359(2):119-129.

31. Kilewo C, Karlsson K, Ngarina M, et al. Prevention of mother-to-child transmission of HIV-1 through breastfeeding by treating mothers with triple antiretroviral therapy in Dar es Salaam, Tanzania: the Mitra Plus study. *J Acquir Immune Defic Syndr.* 2009;52(3):406-416.

32. West NS, Schwartz SR, Yende N, et al. Infant feeding by South African mothers living with HIV: implications for future training of health care workers and the need for consistent counseling. *Int Breastfeed J.* 2019;14:11.

33. UNICEF. Breastfeding A mother's Gift for Every Child. Published 2018. Accessed January 16, 2020.

34. Institute for Health Metrics and Evaluation. Global Burden of Disease Estimates. <http://ghdx.healthdata.org/gbd-results-tool>. Published 2017. Accessed 2019.

35. Newell ML, Coovadia H, Cortina-Borja M, et al. Mortality of infected and uninfected infants born to HIV-infected mothers in Africa: a pooled analysis. *Lancet.* 2004;364(9441):1236-1243.

36. Kim SH, Gerver SM, Fidler S, Ward H. Adherence to antiretroviral therapy in adolescents living with HIV: systematic review and meta-analysis. *AIDS.* 2014;28(13):1945-1956.

37. UNAIDS AIDSinfo. <https://aidsinfo.unaids.org/>. Published 2019. Accessed March 19, 2020.

38. Woldesenbet SA, Kufa T, Lombard C, et al. The 2017 National Antenatal Sentinel HIV Survey, South Africa, National Department of Health. 2019.

39. Ronen K, McGrath CJ, Langat AC, et al. Gaps in Adolescent Engagement in Antenatal Care and Prevention of Mother-to-Child HIV Transmission Services in Kenya. *J Acquir Immune Defic Syndr.* 2017;74(1):30-37.

40. Prachniak-Rincon C, Villar de Onis J. HIV and the Right to Health in Colombia. *Health Hum Rights.* 2016;18(2):157-169.

41. Cintron C, Mudhune V, Haider R, et al. *Costs of HIV Viral Load and Early Infant Diagnosis Testing in Kenya.* Health, Finance & Governence; USAID;2017.

42. Larson BA, Bii M, Halim N, Rohr JK, Sugut W, Sawe F. Incremental treatment costs for HIV-infected women initiating antiretroviral therapy during pregnancy: A 24-month micro-costing cohort study for a maternal and child health clinic in Kenya. *PloS one.* 2018;13(8):e0200199.

43. Meyer-Rath G, van Rensburg C, Chiu C, Leuner R, Jamieson L, Cohen S. The per-patient costs of HIV services in South Africa: Systematic review and application in the South African HIV Investment Case. *Plos One.* 2019;14(2):e0210497.

44. The World Bank. *Ukraine HIV Program Efficiency Study: Can Ukraine improve value for money in HIV service delivery?* Washington, DC2013.

45. Bautista-Arredondo S, Sosa-Rubí SG, Opuni M, et al. Costs along the service cascades for HIV testing and counselling and prevention of mother-to-child transmission. *AIDS.* 2013;30(16):2495-2504.

46. Roberts DA, Barnabas RV, Abuna F, et al. The role of costing in the introduction and scale-up of HIV pre-exposure prophylaxis: evidence from integrating PrEP into routine maternal and child health and family planning clinics in western Kenya. *J Int AIDS Soc.* 2019;22 Suppl 4:e25296.

47. Alistar SS, Owens DK, Brandeau ML. Effectiveness and cost effectiveness of oral pre-exposure prophylaxis in a portfolio of prevention programs for injection drug users in mixed HIV epidemics. *PloS one.* 2014;9(1):e86584.

48. CDC, Kenya Ministry of Health. *The Cost of Comprehensive HIV Treatment in Kenya.* 2013.

49. *South Africa National Department of Health. National HIV Testing Services: Policy.* 2016.

50. U.S. Bureau of Labor Statistics. CPI Inflation Calculator. <https://www.bls.gov/data/inflation_calculator.htm>. Accessed December 15, 2017.
