# Supplementary material for: Optimizing HIV retesting during pregnancy and postpartum in four countries: a cost‐effectiveness analysis
Source: J Int AIDS Soc. 2021 Mar 31;24(4):e25686. doi: 10.1002/jia2.25686 (PMC8010369; doi:10.1002/jia2.25686)
Supplement: Supplementary file 5 — Appendix S4. Comparison of model vs published estiamtes of maternal to child transmission [file JIA2-24-e25686-s006.docx]

Appendix 4: Comparison of model vs published estiamtes of maternal to child transmission

| **Country** | **Scenario** | **Model estimate** | **Published estimate** |
| --- | --- | --- | --- |
| Kenya | National guidelines*  No retesting  Late ANC | 7.6%  9.5%  7.7% | 8.9% (1) |
| South Africa | National guidelines* | 6.1% | 4.6% (2) |
|  | No retesting | 7.1% |  |
|  | Late ANC | 6.2% |  |
| Colombia | National guidelines* | 5.7% | 3.8% (3) |
|  | No retesting | 6.8% |  |
|  | Late ANC | 5.7% |  |
| Ukraine | National guidelines* | NA | 3.7% (4) |
|  | No retesting | 2.6% |  |
|  | Late ANC | 2.5% |  |

**Table A4:** Modeled versus published estimates of maternal to child transmission. Model estimates are derived by dividing the total number of HIV+ infants at 12 months postpartum by the total number of HIV+ mothers at 12 months postpartum. *Kenya: Late pregnancy/delivery, six weeks postpartum, six months postpartum (5); South Africa: each antenatal care visit and every three months during pregnancy and while breastfeeding (6); Colombia: each trimester and at delivery (7); Ukraine: no guidelines.

**References**

1. McGrath CJ, Singa B, Langat A, Kinuthia J, Ronen K, Omolo D, et al. Non-disclosure to male partners and incomplete PMTCT regimens associated with higher risk of mother-to-child HIV transmission: a national survey in Kenya. AIDS Care. 2017:1-9.

2. Johnson LF, May MT, Dorrington RE, Cornell M, Boulle A, Egger M, et al. Estimating the impact of antiretroviral treatment on adult mortality trends in South Africa: A mathematical modelling study. PLoS Med. 2017;14(12):e1002468.

3. Elimination of mother-to-child transmission of HIV and syphilis in the Americas. Update 2016. Washington, D.C. : PAHO; 2017.

4. Bozicevic ID, Z. Report on Pre-Validation of Elimination of Mother-to-Child Transmission of HIV in Ukraine. December 2018. .

5. Ministry of Health, National AIDS & STI Control Programme. Guidelines on use of Antiretroviral Drugs for Treating and Preventing HIV Infection in Kenya 2016. Nairobi, Kenya: NASCOP; July 2016.

6. National Department of Health. National HIV Testing Services: Policy. Republic of South Africa: Department of Health. 2016.

7. ETMI - PLUS: Estrategia Nacional para la Eliminación de la Transmisión Materno Infantil del VIH, la sífilis congénita, la hepatitis B y la enfermedad de Chagas. Comportamiento de la Transmisión Materno Infantil del VIH en Colombia. Medición de la Cohorte 2017. Dirección de Promoción y Prevención Grupo de Sexualidad, Derechos Sexuales y Derechos Reproductivos. Colombia 29 de mayo de 2019
