# Supplementary material for: Optimizing HIV retesting during pregnancy and postpartum in four countries: a cost‐effectiveness analysis
Source: J Int AIDS Soc. 2021 Mar 31;24(4):e25686. doi: 10.1002/jia2.25686 (PMC8010369; doi:10.1002/jia2.25686)
Supplement: Supplementary file 6 — Appendix S5. Microcosting methods [file JIA2-24-e25686-s001.docx]

Appendix 5: Microcosting methods

We conducted a microcosting study of routine HIV testing in maternal and child health facilities in Kenya. This costing analysis was nested in an ongoing study implementing repeat maternal testing during HIV and pregnancy using a 4^th^ generation screening test. We collected cost data between June and November 2017. We conducted time and motion studies to estimate personnel costs, and we abstracted the costs of HIV test kits and other testing supplies from clinic registers at Ahero County and Bondo sub-County Hospitals in western Kenya. We analyzed costs from the provider perspective and excluded research-related costs. We used the resource utilization data and commodity prices from this study to estimate costs of certain input parameters (see Appendix 3).
