# Supplementary material for: Optimizing HIV retesting during pregnancy and postpartum in four countries: a cost‐effectiveness analysis
Source: J Int AIDS Soc. 2021 Mar 31;24(4):e25686. doi: 10.1002/jia2.25686 (PMC8010369; doi:10.1002/jia2.25686)
Supplement: Supplementary file 7 — Appendix S6. Infections averted under optimized MCH attendance and HIV care cascade [file JIA2-24-e25686-s004.docx]

Appendix 6: Infections averted under optimized MCH attendance and HIV care cascade

To assess how maternal retesting benefits would change with improved MCH attendance and optimal HIV care cascade parameters, we increased the following parameters to 100%: attendance at each antenatal and postnatal care visit, HIV test coverage, ART initiation, viral suppression, retention in ART care, and infant ARV prophylaxis. Under a strategy of retesting in late ANC as well as every three months postpartum (scenario 7), we compared the percentage of maximum infant infections averted through retesting (excluding mothers with known HIV-positive status prior to pregnancy) using the optimized parameters rather than the literature parameters. With higher ANC attendance and HIV care cascade parameters, more infections are averted through testing at first ANC, which decreases the maximum number of potential infections that could be averted through retesting strategies. However, the percentage of the maximum number of potential infections averted through retesting does not change substantially with optimized parameters compared to literature parameters.

**Table A6:** Impact of MCH and HIV care cascade parameters on the number of infant infections averted under scenario 7 compared to scenario 1 (no retesting), expressed as a percentage of the total number of infections that could potentially be averted using retesting strategies. ^†^Total number of infant infections under retesting in late ANC as well as every three months postpartum (scenario 7) subtracted from total number of infant infections under no retesting (scenario 1). ^‡^Number of infant infections under no retesting after excluding mothers with known HIV-positive status prior to pregnancy.

|  | **Literature Parameters** | | | **Optimized Parameters** | | |
| --- | --- | --- | --- | --- | --- | --- |
| **Country** | # infections averted^†^ | Max potential # infections averted^‡^ | % | # infections averted^†^ | Max potential # infections averted^‡^ | % |
| Kenya | 2,982 | 11,135 | 27 | 1,580 | 5,857 | 22 |
| South Africa | 3,562 | 16,660 | 21 | 1,805 | 9,334 | 19 |
| Colombia | 31 | 115 | 27 | 22 | 93 | 24 |
| Ukraine | 5 | 17 | 32 | 6 | 16 | 34 |
